# 5‐Hydroxymethylcytosine Dynamics Reveals Coordinated Reprogramming of Parental Genomes and X Chromosome Dosage Balance in Mouse SCNT Embryos

**DOI:** 10.1002/advs.202509682

**Published:** 2025-11-18

**Authors:** Zeming Xiang, Rui Yan, Jing Guo, Mengyao Wang, Xin Cheng, Fan Zhang, Tianzi Guo, Xin Long, Fan Guo, Dan Liang

**Affiliations:** ^1^ Department of Obstetrics and Gynecology NHC Key Laboratory of Study on Abnormal Gametes and Reproductive Tract The First Affiliated Hospital of Anhui Medical University Hefei 230022 China; ^2^ State Key Laboratory of Organ Regeneration and Reconstruction Institute of Zoology University of Chinese Academy of Sciences Chinese Academy of Sciences Beijing 100101 China; ^3^ Beijing Institute for Stem Cell and Regenerative Medicine Beijing 100101 China

**Keywords:** 5‐hydroxymethylcytosine, embryonic development, epigenetic barrier, somatic cell nuclear transfer

## Abstract

Somatic cell nuclear transfer (SCNT) embryos exhibit widespread epigenetic defects, particularly aberrant DNA methylation. DNA 5‐hydroxymethylcytosine (5hmC) is involved in methylation reprogramming during early embryonic development, yet its role in SCNT embryos remains largely unknown. Here, the genome‐wide 5hmC landscapes in mouse SCNT embryos are systematically profiled with parental allele specificity. It is revealed that both maternal and paternal genomes of donor somatic cells acquire a transient, sperm‐like but attenuated and allele symmetric distribution of 5hmC at the 2‐cell stage, distinct from the parental asymmetric pattern observed in naturally fertilized eggs. This is characterized by insufficient DNA hydroxymethylation of the X chromosome in female SCNT embryos, as well as resistance to 5hmC‐associated DNA demethylation at germline imprinting control regions (gICRs). While de novo 5hmC generation is closely associated with initial DNA demethylation during somatic‐to‐zygotic transition, it later becomes uncoupled from ongoing methylation changes. Importantly, global elevation of 5hmC via Tet3 overexpression leads to premature activation of developmental genes at the 2‐cell stage and severely impairs SCNT embryo development. These findings reveal unique dynamics and functional consequences of abnormal 5hmC remodeling in SCNT embryos, highlighting the precise regulation of 5hmC generation as a key epigenetic event for successful mammalian cloning.

## Graphical abstract

Genome‐wide, high‐resolution profiling of hydroxymethylation in mouse SCNT embryos reveals a transient, allele‐symmetric 5hmC reprogramming pattern distinct from natural embryos, with X‐chromosomes and imprinting control regions resistant to proper remodeling. Ectopic generation of 5hmC prematurely activates developmental genes and impairs embryogenesis, highlighting the necessity of both precise levels and correct distribution of hydroxymethylation for successful mammalian cloning.

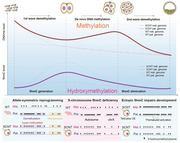

## 1 Introduction

Mouse somatic cell nuclear transfer (SCNT), also known as cloning, was developed based on the fact that mammalian oocytes are capable of reprogramming the transferred somatic cell nuclei into a totipotent zygotic state that enables the subsequent embryogenesis.^[^ ^1^, ^2^, ^3^ ^]^ Such somatic‐to‐zygotic transition (SZT) involves the remodeling of complicated epigenetic processes including histone variants incorporation,^[^ ^4^, ^5^ ^]^ chromatin structure,^[^ ^6^ ^]^ histone modifications,^[^ ^7^, ^8^, ^9^ ^]^ X chromosome inactivation,^[^ ^10^, ^11^, ^12^ ^]^ DNA methylation reprogramming,^[^ ^9^, ^13^, ^14^, ^15^ ^]^ etc. Beyond its application in agricultural biotechnologies and endangered species conservation, SCNT provides an ideal model to investigate how terminally differentiated somatic cells are reprogrammed into embryonic cells by cytoplasmic maternal factors in the oocyte and how totipotency is established. Though various perturbations were developed to overcome the epigenetic barriers of SCNT embryos,^[^ ^7^, ^9^, ^10^, ^12^, ^15^, ^16^ ^]^ the detailed landscape of the epigenome, especially the DNA methylome and hydroxymethylome in SCNT embryos still requires comprehensive elucidations.

DNA 5‐hydroxymethylcytosine (5hmC), a DNA modification derived from 5‐methylcytosine (5mC), plays a crucial role in regulating gene expression and epigenetic reprogramming during mammalian development.^[^ ^13^ ^]^ Generated by the ten‐eleven translocation (TET) family enzymes (Tet1/2/3), emerging evidences highlighted 5hmC's broader functions in germ cells, early embryos, and neurons beyond a transient intermediate in active DNA demethylation.^[^ ^17^, ^18^, ^19^, ^20^, ^21^, ^22^ ^]^ Notably, 5hmC is dynamically regulated during early embryogenesis, particularly at the zygote‐to‐blastocyst development.^[^ ^23^, ^24^ ^]^ Tet3‐mediated hydroxymethylation was reported to play a pivotal role in the epigenetic reprogramming of the paternal genome shortly after fertilization.^[^ ^13^ ^]^ Genome‐wide profiling studies revealed that 5hmC is predominantly enriched in regulatory regions, such as enhancers, where it facilitates transcriptional activation and chromatin remodeling.^[^ ^17^, ^25^, ^26^, ^27^ ^]^ Despite our recent studies having uncovered the biological functions of 5hmC in the development of fertilized mouse and human embryos,^[^ ^24^, ^28^ ^]^ the role of DNA hydroxymethylation in embryogenesis of SCNT mouse model remains poorly characterized.

In this study, we utilized cutting‐edge, low‐input 5hmC sequencing methods in combination with somatic cells from C57BL/6J × PWK/Phj background mice as nuclear donors to characterize the dynamics of DNA hydroxymethylation during the development of SCNT embryos. For the first time in SCNT mouse model, we elucidated the relationship between the dynamics of 5mC and 5hmC, compared the 5hmC configurations between autosomes and the X chromosome, and explored the epigenome dynamics of germline imprinting control regions (gICRs). We also analyzed the epigenome of SCNT embryos with parental alleles separately, showing 5hmC profile between maternal and paternal genomes in cloned embryos. Additionally, gain‐of‐function approaches were introduced to further identify the effect of ectopically generated 5hmC on SCNT embryogenesis. Overall, our study demonstrated a comprehensive landscape and dynamics of 5hmC in mouse SCNT embryos, providing systematic resources with mechanistic insights into the epigenetic reprogramming driven by maternal factors in ooplasm during the cloning of mammals.

## 2 Results

### 2.1 Landscape of 5hmC in SCNT Embryos Across Developmental Stages

**Figure 1:**
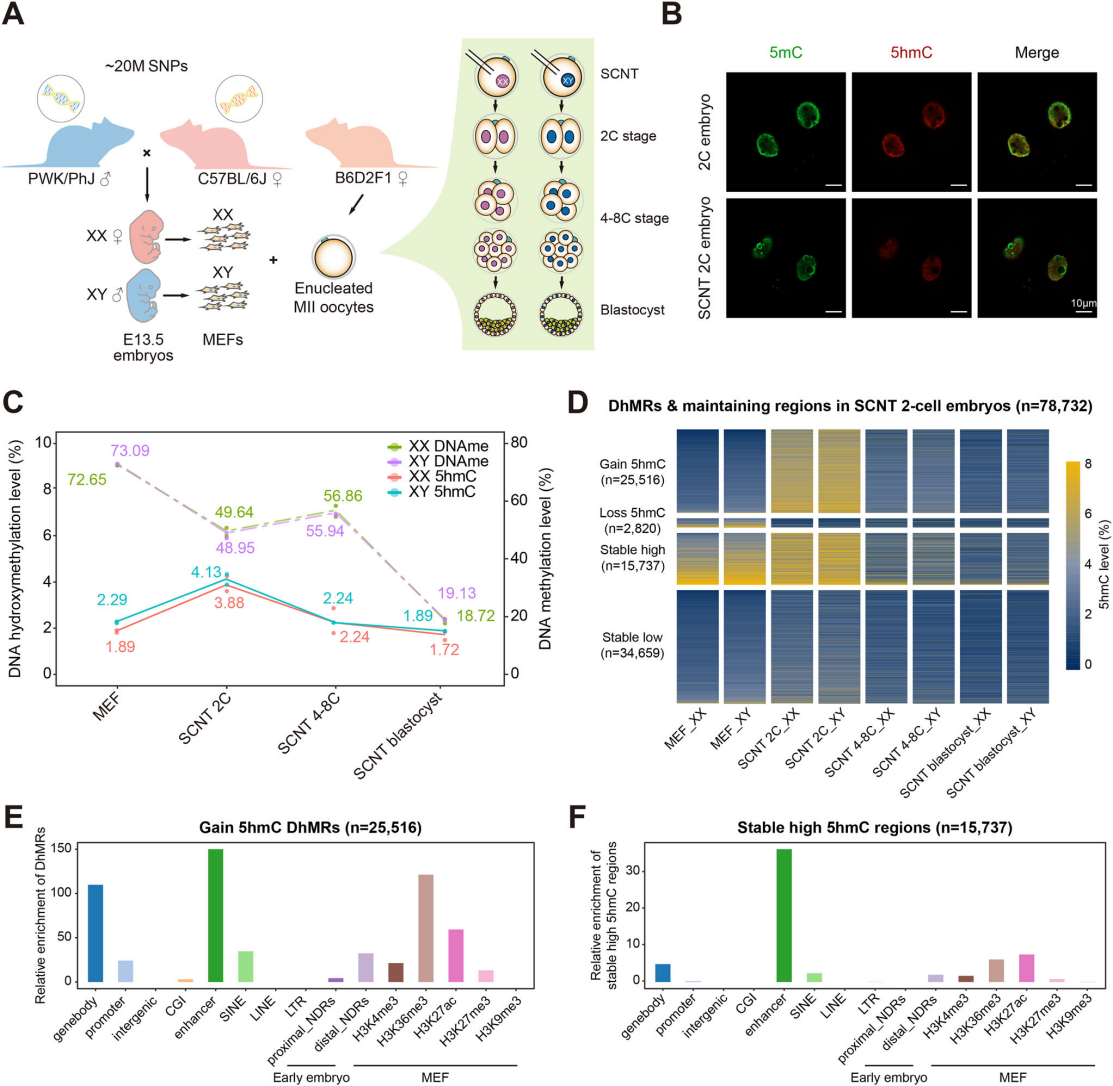
Global 5hmC dynamics and landscape in SCNT nucleus across developmental stages. A) Schematic illustration of experimental approaches. Embryos generated by SCNT were used for whole‐genome bisulfite sequencing (WGBS), APOBEC‐coupled epigenetic sequencing (ACE‐seq), or RNA‐seq. B) Immunostaining of 5mC and 5hmC in WT and SCNT 2‐cell embryos. C) The dynamics of 5hmCpG and DNA methylation levels in donor cells (MEFs) and SCNT preimplantation embryos across developmental stages. (*n* = 2 biological replicates for each gender at every stage, as shown in Table S1, Supporting Information). D) Heatmaps showing dynamics of 5hmCpG levels in mouse MEFs and SCNT preimplantation embryos across developmental stages. Four categories of genomic regions were identified based on the changes in 5hmC level during MEF‐to‐SCNT 2‐cell transition. Differentially hydroxymethylated regions (DhMRs) were calculated with data of male and female embryos merged together, and the 5hmC levels were displayed separately by gender. E,F) Bar plots showing the enrichment scores of gain 5hmC DhMRs (E) and stable high 5hmC regions (F) across various genomic elements. Mouse MEF histone modification datasets are extracted from Gene Expression Omnibus (GEO) database under accession number GSE73124, GSE90893 and GSE169635.

To accurately investigate the dynamics of 5hmC across various developmental stages, we employed single‐base resolution, low‐input APOBEC‐coupled epigenetic sequencing (ACE‐seq), together with whole‐genome bisulfite sequencing (WGBS), on SCNT mouse embryos reconstructed from male and female mouse embryonic fibroblasts (MEFs) derived from C57BL/6J female × PWK/Phj male hybrid mice, which possess distinguishable parental SNPs. SCNT embryos at 2‐cell, 4 to 8‐cell, and blastocyst stages were collected and analyzed for each sex, with two biological replicates per stage (**Figure** **1**A), which represents the accepted standards in studies of early embryonic epigenomes. Each replicate contained at least 200 cells, with an average sequencing depth of 92 Gb and an average genome coverage of 82%. A total of 3364 Gb raw sequencing data were generated in this study. The quality of both ACE‐seq and WGBS datasets was rigorously assessed (Figure S1 and Table S1, Supporting Information), ensuring high data quality and reproducibility and providing a reliable basis for subsequent genome‐wide analyses. Strikingly, we observed a marked elevation in global 5hmC levels at the 2‐cell stage (Figure 1B,C). Subsequently, the global 5hmC level gradually decreased in SCNT embryos at the 4–8 cell stages, reaching the lowest level in blastocysts (Figure 1C). More specifically, during the somatic‐to‐zygotic nuclear transition (SZT), SCNT embryos of both sexes exhibited a substantial increase in the proportion of high‐5hmCpG tiles across the genome (Figure S2A,B, Supporting Information), and in 5hmC levels around gene bodies and flanking regions (excluding transcription start sites, TSSs) (Figure S2C, Supporting Information). These levels peaked at the 2‐cell stage before progressively declining toward the blastocyst stage (Figure S2, Supporting Information). In contrast, genomic DNA methylation in cloned embryos rapidly decreased from activation to the 2‐cell stage, rebounded between the 2‐cell and 4 to 8‐cell stages, and then decreased again until the blastocyst stage (Figure 1C), which is consistent with previous reports.^[^ ^15^ ^]^ We identified 25516 loci undergoing 5hmC generation, 2820 loci showing 5hmC loss, and 50396 loci inheriting either high (15737) or low (34659) 5hmC levels from MEF genomes during SZT reprogramming. All these four categories of genomic regions exhibited subsequent 5hmC reduction until the SCNT blastocyst stage (Figure 1D). Notably, loci with either 5hmC generation or maintained high 5hmC levels were preferentially enriched in enhancer regions, gene bodies, and genomic regions modified by H3K36me3, H3K27ac, or H3K4me3 (Figure 1E,F). In summary, 5hmC generation predominantly occurs during the SZT transition at transcriptionally active regulatory elements in SCNT embryos, exhibiting a “up‐down” dynamic pattern prior to implantation. Meanwhile, DNA methylation dynamics followed a “demethylation‐de novo methylation‐demethylation” pattern, reflecting a two‐wave demethylation process in SCNT mouse embryos. No significant differences were detected in global levels of either 5hmC or 5mC between pre‐implantation SCNT embryos derived from nuclear donors of different sexes (XX or XY).

Noting that both gain 5hmC differentially hydroxymethylated regions (DhMRs) and stable high 5hmC regions were preferentially enriched at enhancers, we next investigated their characteristics during the MEF‐to‐SCNT 2C transition. We identified 22 869 enhancers carrying both features, corresponding to 2691 genes (Figure S3A, Supporting Information). Based on this, enhancers were classified into three groups: newly generated 5hmC‐associated enhancers (NGEs), stable high 5hmC‐associated enhancers (SHEs), and bivalent enhancers (BVEs) containing both. Sequence features such as CpG density and GC content were similar across groups (Figure S3B, Supporting Information). During the MEF‐to‐SCNT 2C transition, NGEs displayed strong 5hmC elevation, whereas SHEs started with higher basal 5hmC levels; all three groups underwent comparable DNA demethylation driven by global reprogramming (Figure S3C, Supporting Information). Gene expression level linked to these enhancers showed no overall differences (Figure S3D, Supporting Information). Notably, although <2% of enhancers in each group overlapped with known ZGA enhancers, these accounted for >90% of total ZGA enhancers, with NGEs contributing the majority (Figure S3E, Supporting Information). ZGA genes associated with BVEs were expressed most highly, followed by those linked to NGEs and SHEs (Figure S3F, Supporting Information). To summarize, these findings suggest that while NGEs, SHEs, and BVEs share broadly similar sequence properties, their distinct 5hmC dynamics shape enhancer activity in different ways, pointing to specialized regulatory roles during SCNT reprogramming.

### 2.2 5hmC Contributes to the First Wave DNA Demethylation in SCNT Embryos

**Figure 2:**
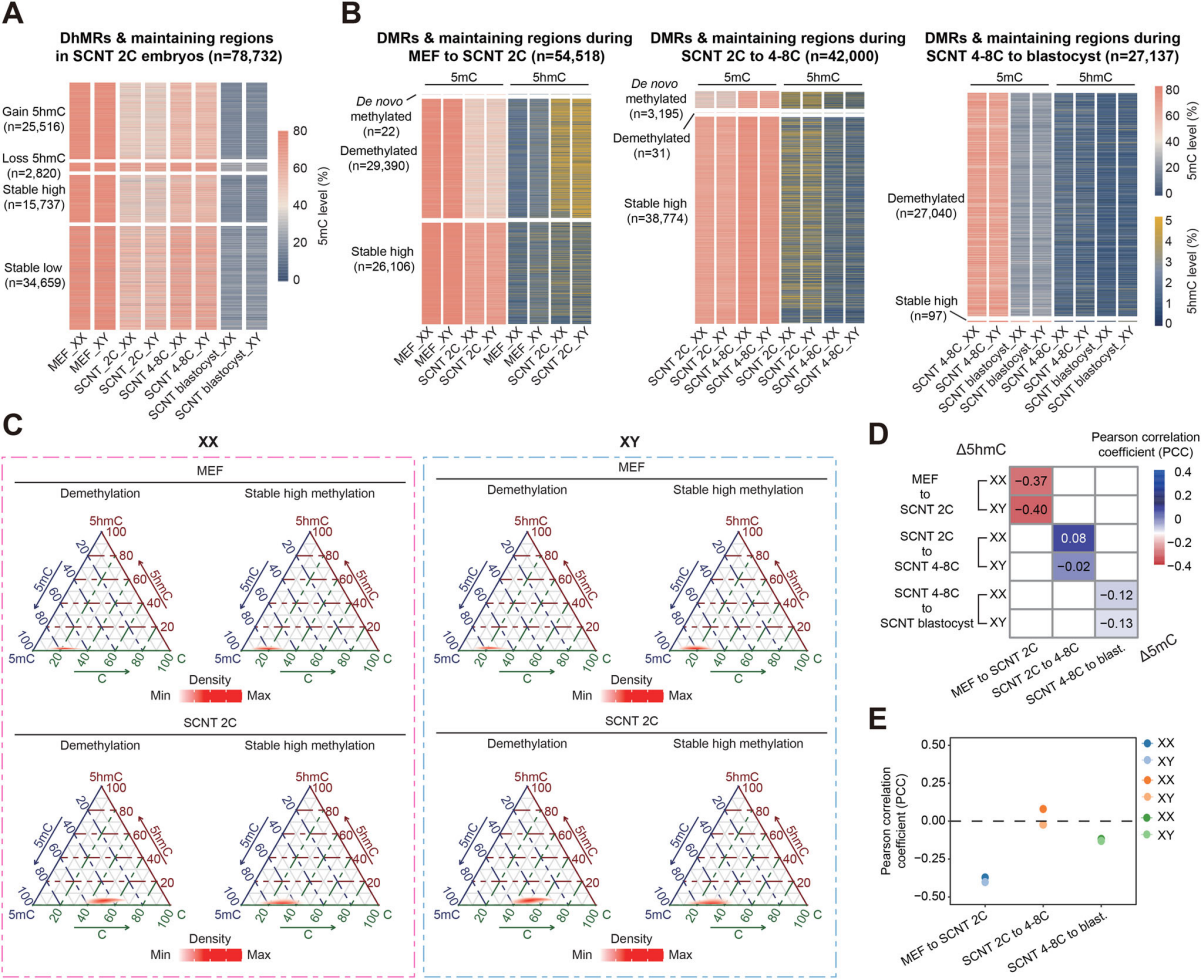
Correlation between the dynamics of DNA hydroxymethylation and DNA methylation before SCNT blastocyst. A) Heatmaps showing the dynamics of 5mC levels in genomic regions identified in Figure 1D based on 5hmC level change during MEF‐to‐SCNT 2‐cell embryo transition. B) Heatmaps displaying 5mC level and 5hmC level in differentially methylated regions (DMRs) at each developmental stage: MEF to SCNT 2‐cell stage (left), SCNT 2‐cell to 4 to 8‐cell stage (middle), and SCNT 4 to 8‐cell to blastocyst stage (right). C) Ternary plots showing the levels of cytosine, 5mC, and 5hmC in demethylated DMRs and maintenance regions in female (left) and male (right) cloned embryos. D,E) Heatmaps (D) and dot plot (E) illustrating the correlation between changes in 5hmC and 5mC levels of the DMRs during each developmental stage of SCNT mouse embryo, calculated in Pearson correlation coefficients (PCC). (For MEF‐to‐SCNT 2C, *n* = 54 518; for SCNT 2C to 4‐8C, *n* = 42 000; for SCNT 4‐8C to blastocyst, *n* = 27 137).

We next explored the relationship between 5mC and 5hmC in the genome of SCNT pre‐implantation embryos across various developmental stages. Across all four categories of DhMRs or 5hmC maintaining regions defined above, we observed that the dynamics of 5mC from somatic cells to SCNT blastocysts were consistent with the global DNA methylation patterns (**Figure** **2**A). In the reversed analysis, genomic regions were categorized based on 5mC variations at each developmental stage from somatic cell to SCNT blastocyst in both male and female embryos, and then the corresponding 5hmC dynamics within these region types were evaluated. Notably, demethylated differentially methylated regions (DMRs) during SZT undergo significant 5hmC generation simultaneously, whereas the stable DNA methylation regions exhibited no detectable 5hmC increase in either male or female SCNT 2‐cell embryos (Figure 2B,C). Interestingly, during the subsequent de novo methylation and second‐wave demethylation events from 2‐cell to blastocyst stage, 5hmC levels remained comparable across genomic regions undergoing distinct DNA methylation dynamics (Figure 2B; Figure S4, Supporting Information). Quantitative analysis of correlations between changes of 5hmC and 5mC at each developmental stage further confirms that DNA methylation and hydroxymethylation display a significant negative correlation during the MEF‐to‐SCNT 2‐cell stage compared to other developmental stages (Figure 2D,E). Collectively, these results suggest that although 5hmC variation does not necessarily induce 5mC changes, the first‐wave DNA demethylation is closely linked with 5hmC generation exclusively during somatic‐to‐zygotic transition in both male and female mouse SCNT embryos. In contrast, the subsequent de novo methylation and second‐wave demethylation display no observable coupling with 5hmC dynamics.

### 2.3 5hmC Dynamics in Germline Imprinting Control Regions of SCNT Embryos

**Figure 3:**
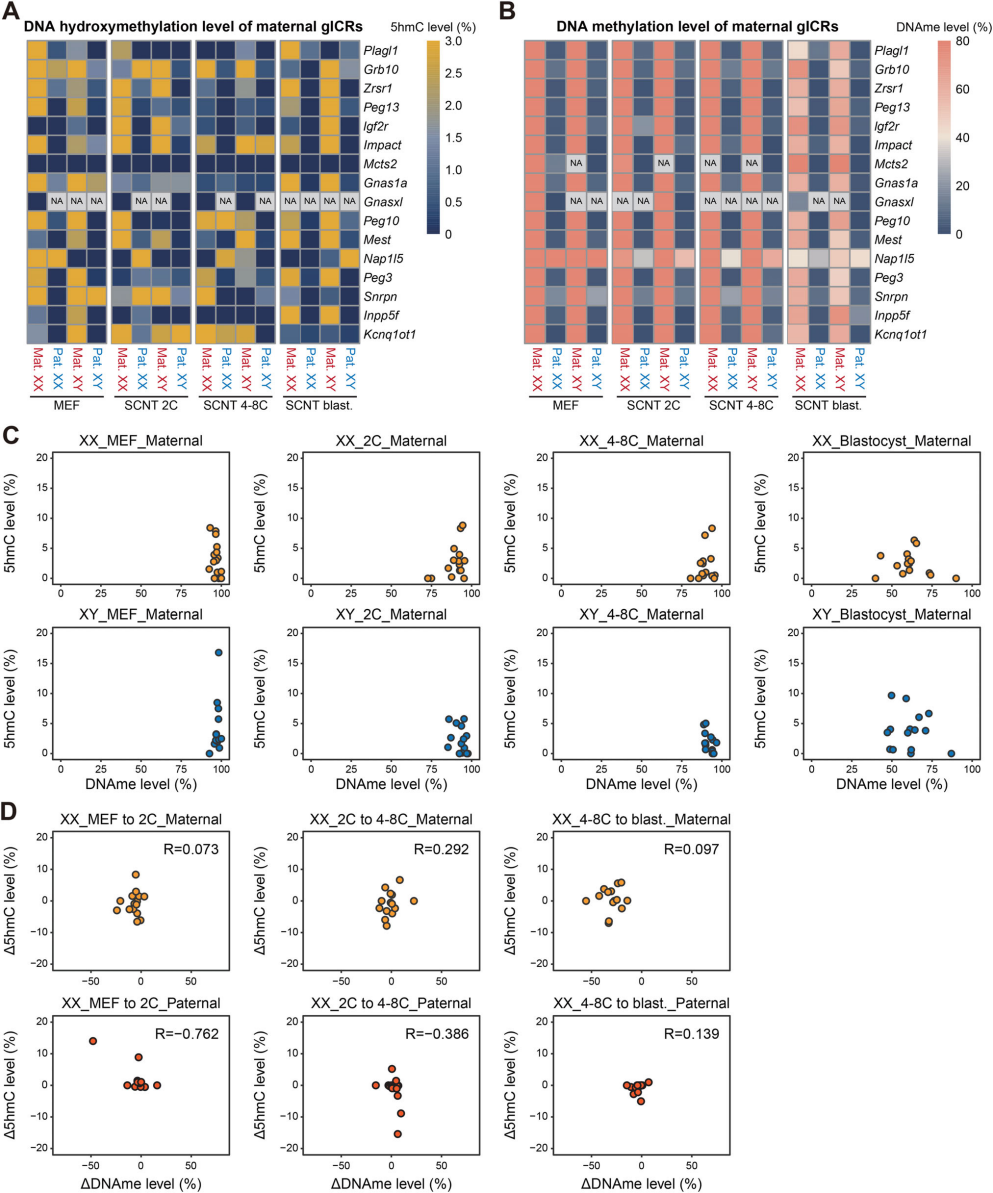
5hmC level on maternal gICRs across SCNT developmental stages. A,B) Heatmaps showing the dynamics of DNA hydroxymethylation (A) and methylation (B) levels at maternal germ‐line imprinting control regions (M_gICRs) in both parental genomes in SCNT embryos across developmental stages. (*n* = 2 biological replicates for each gender at every stage). C) Dot plot of DNA methylation and 5hmC levels at the same M_gICRs loci in the maternal genome. M_gICRs in female and male SCNT embryos are colored yellow and blue, respectively. D) Dot plot of DNA methylation and 5hmC level changes between each 2 developmental stages at the same M_gICRs loci in the maternal genome. M_gICRs in female and male SCNT embryos are colored yellow and blue, respectively.

As a distinct class of genomic regions exhibiting different methylation dynamics on parental alleles during early embryogenesis, the DNA methylation defects of germline imprinting control regions (gICRs) were implicated in developmental abnormalities of SCNT embryos in previous studies.^[^ ^9^ ^]^ The observed relationship above between 5mC and 5hmC prompted us to further dissect the role of 5hmC dynamics in DNA methylation aberrations at gICRs in SCNT embryos. Given the limited number of known paternal gICRs in mice and the insufficient DNA hydroxymethylation data available for these regions, we focused our analysis exclusively on the 17 well‐characterized maternal gICRs. We first examined both 5hmC and DNA methylation level at 17 maternal gICRs across both parental alleles at each developmental stage, from somatic cells to SCNT blastocysts of both sexes (**Figure** **3**A,B). Though maternal gICRs remained hyper‐methylated until the SCNT 4‐8 cell stage, significant methylation loss occurred at most gICRs on maternal alleles in both XX and XY SCNT blastocysts during the second wave of demethylation (Figure 3B). Intriguingly, this demethylation process was not coupled with substantial accumulation of 5hmC (Figure 3A). Unlike the global “up‐down” pattern of 5hmC dynamics, the 5hmC level of gICRs did not exhibit significant fluctuations during the pre‐implantation development (Figure S5A, Supporting Information). Notably, unlike the strong association of global 5hmC generation and first‐wave DNA demethylation during MEF to SCNT 2‐cell stage, the 5hmC level and DNA methylation level of maternal gICRs show minimal correlation across every developmental stage during early embryogenesis, regardless of parental origin or embryo sex (Figure 3C,D; Figure S5B,C, Supporting Information). These results suggested that the gICRs on maternal alleles undergo significant loss of imprinting during the second wave of demethylation in SCNT blastocyst in a 5hmC‐independent manner.

### 2.4 5hmC Accumulation on the X Chromosome in Parental Genomes of SCNT Embryos

**Figure 4:**
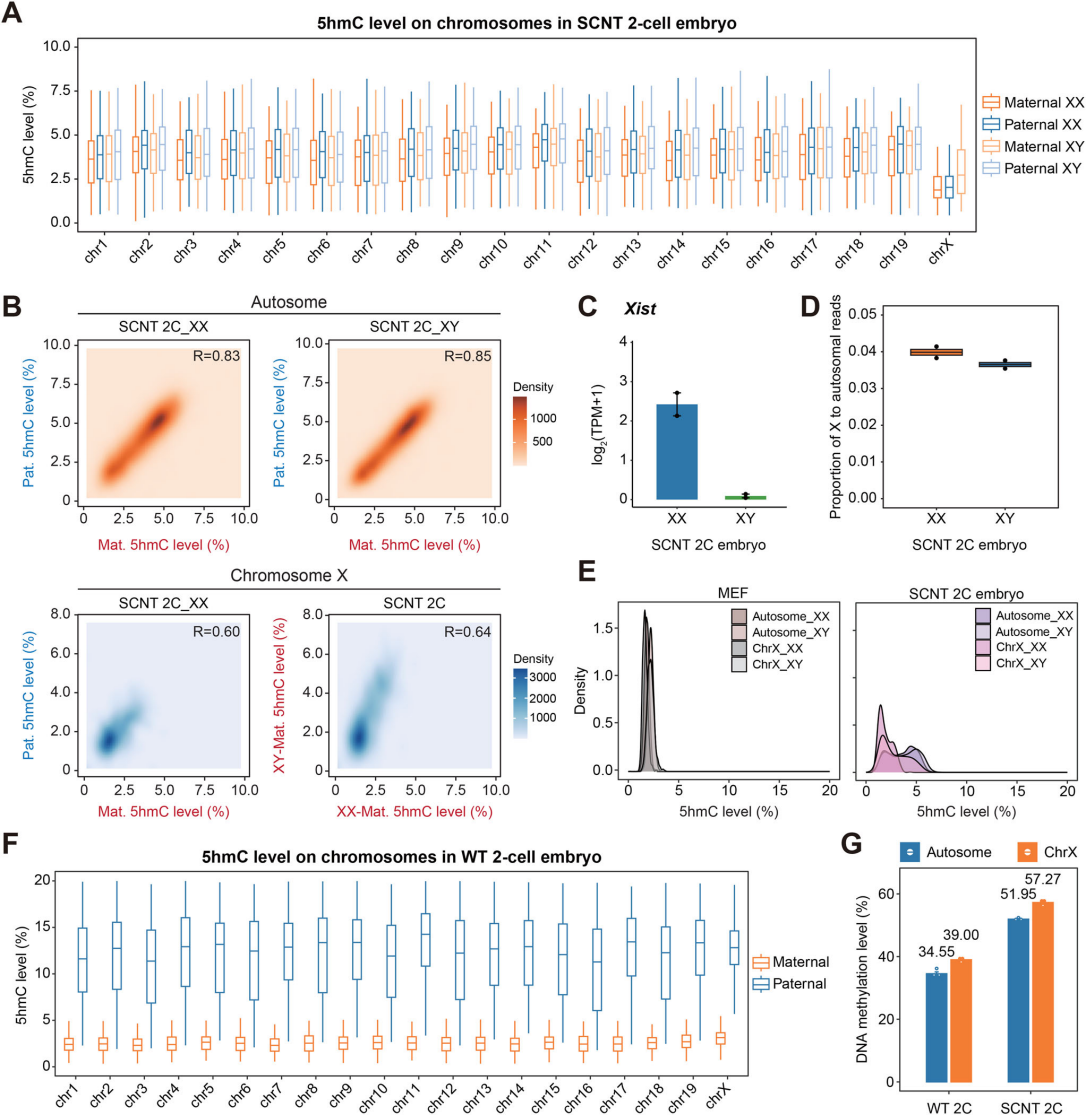
The 5hmC distribution difference between male and female embryos and between X chromosome and autosomes. A) Box plots showing 5hmC levels in 19 autosomes and the X chromosome in maternal and paternal genomes from SCNT embryos of both genders (*n* = 2 biological replicates for each gender). B) Density plots comparing 5hmC levels in autosomes (top) and the X chromosome (bottom) between the parental genomes. C) Bar plots illustrating the expression level of the Xist gene in both XX and XY SCNT 2‐cell embryos. Error bars represent mean ± s.e.m (*n* = 2 biological replicates for each gender). D) Proportion of X chromosome transcript reads relative to autosomal transcript reads in SCNT 2‐cell embryos of both genders (*n* = 2 biological replicates for each gender). E) Density plots showing the distribution of 5hmC levels in genomic tiles on autosomes and the X chromosome in MEFs and SCNT 2‐cell embryos. F) Box plots of chromosome‐wise 5hmC level in both parental genomes of WT 2‐cell embryos (*n* = 2 biological replicates for each gender). G) Bar plots displaying the DNA methylation levels of both autosomes and the X chromosome in WT and SCNT 2‐cell embryos (*n* = 2 biological replicates for each gender).

As a dosage compensation mechanism specifically in female genome, X chromosome inactivation (XCI) was characterized as another pivotal epigenetic barrier during SCNT embryonic development.^[^ ^10^ ^]^ In wild‐type female mouse preimplantation embryos, imprinted XCI occurs exclusively on the paternal X chromosome after 2‐cell stage. By contrast, in cloned embryos, Xist is aberrantly activated on maternal X chromosomes, leading to inappropriate silencing of both through DNA methylation–mediated repression. To elucidate how DNA hydroxymethylation is involved in such epigenetic process, we investigated the dynamics of 5hmCpG levels of both autosomes and X chromosomes in the maternal and paternal genomes from SCNT embryos across various developmental stages (**Figure** **4**A; Figure S6A,B, Supporting Information). In both male and female SCNT 2‐cell embryos, comparable 5hmC levels are distributed on maternal and paternal autosomes, while X chromosomes exhibited significantly lower 5hmC levels compared to autosomes. Notably, female embryos displayed even less 5hmC accumulation on both maternal and paternal X chromosomes, relative to the single maternal X chromosome in males (Figure 4A,B). A similar 5hmC distribution pattern persisted through subsequent embryogenesis across all chromosomes, though the difference in 5hmC level gradually diminished with global 5hmC decreasing (Figure S6C, Supporting Information). Consistent with previous studies,^[^ ^10^ ^]^ we observed higher Xist overexpression in female SCNT 2‐cell embryos (Figure 4C). Transcriptome analysis revealed that transcripts from both X chromosomes in female SCNT embryos were equivalent to those from the single X chromosome in males (Figure 4D). Furthermore, comparative analysis with the 5hmC levels in MEF chromosomes ruled out the possibility that the hypo‐hydroxymethylation in X chromosomes of SCNT embryos is inherited from the donor somatic cell (Figure 4E; Figure S6D, Supporting Information). Thus, the gender‐related, X chromosome‐specific low 5hmC accumulation in SCNT embryos predominantly results from insufficient 5hmC generation during the reprogramming at somatic‐to‐zygotic transition. However, such hypo‐5hmC accumulation in X chromosomes was not observed in the WT 2‐cell embryo (Figure 4F). Not surprisingly, the SCNT‐specific 5hmC deficiency on X chromosomes is accompanied by hypermethylation of chrX in SCNT 2‐cell embryos. Nevertheless, the X/A transcript ratio showed no statistically significant difference in gene expression between WT and SCNT embryos at the 2‐cell stage (Figure S6E, Supporting Information). Interestingly, both WT and cloned 2‐cell embryos showed higher DNA methylation levels on X chromosomes than on autosomes (Figure 4G). Given that imprinted X‐chromosome inactivation is initiated between the 4‐ and 8‐cell stages, the findings above suggest that the consequences of insufficient 5hmC deposition on X chromosomes could be expected to manifest at later developmental stages.

### 2.5 Aberrant 5hmC Distribution Links with SCNT Developmental Defects

**Figure 5:**
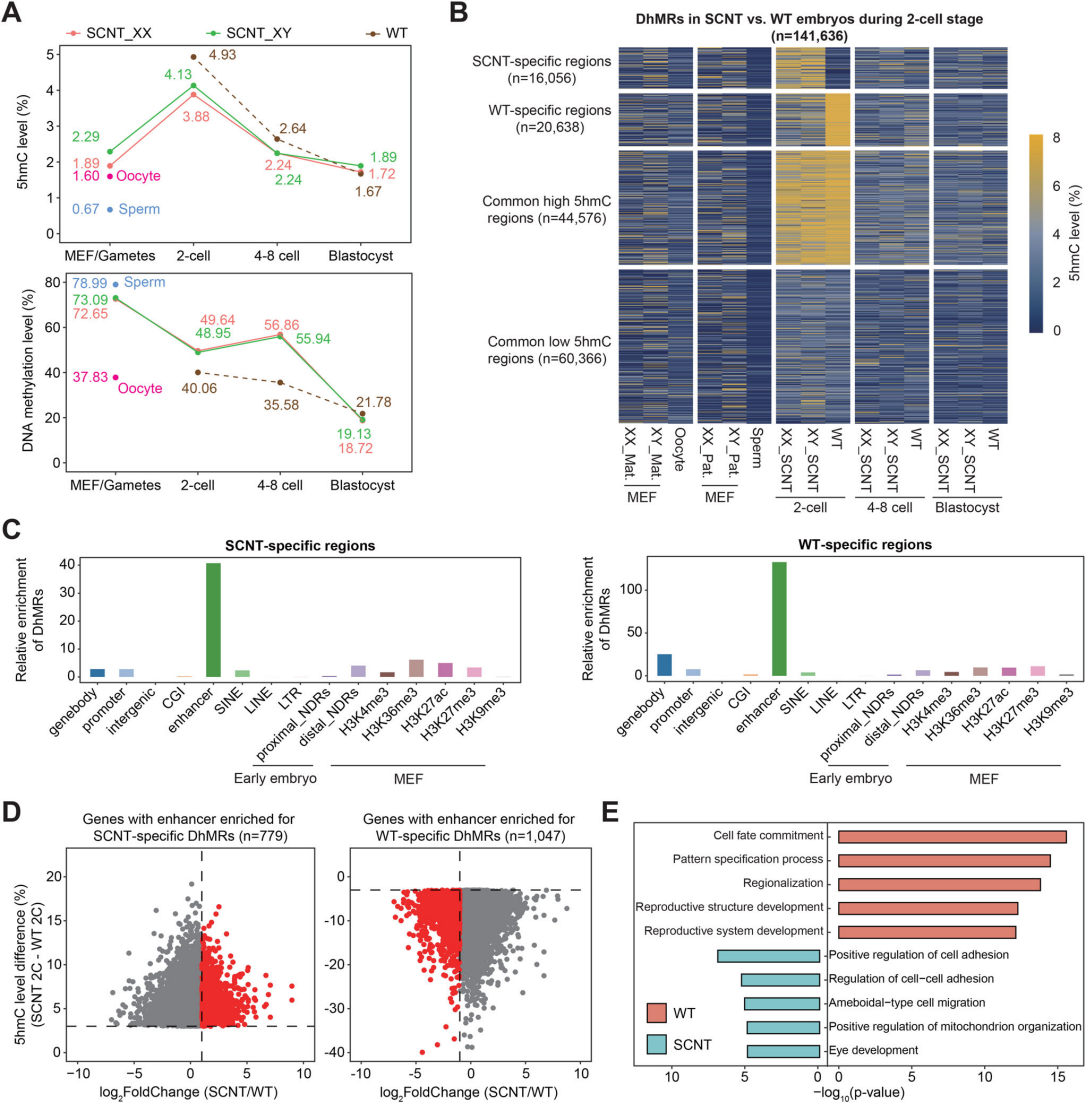
Comparison of 5hmC distribution between WT and SCNT embryos at the 2‐cell stage. A) The dynamics of 5hmCpG (top) and DNA methylation (bottom) levels between somatic cells (MEFs) and gametes, and between SCNT and WT mouse embryos across developmental stages (*n* = 2 biological replicates for each gender at every stage, as shown in Table S1, Supporting Information). B) Heatmaps comparing the 5hmC level dynamics of four kinds of DhMRs/ common regions between WT and SCNT embryos across developmental stages. C) Bar plot showing the enrichment scores of SCNT‐specific 5hmC DhMRs (left) and WT‐specific 5hmC DhMRs (right) across various genomic elements and histone modification regions. D) Scatter plots comparing gene expression level changes (*x*‐axis) and 5hmC level difference (*y*‐axis) of genes related with cell‐type specific enhancers between WT and SCNT 2‐cell embryos. Red dots represent cell‐type‐specific genes with both higher 5hmC level at enhancers and significantly higher expression level in SCNT (left) and WT (right) 2‐cell embryos. E) Bar plots showing Gene Ontology (GO) enrichment analysis of SCNT‐ and WT‐ specific genes identified in Figure 5D.

Multiple epigenetic barriers in SCNT embryos have been identified by comparing with naturally fertilized (WT) embryos, including histone modifications, chromatin accessibility, histone variants, and DNA methylation. However, whether DNA hydroxymethylation differs between WT and SCNT embryos, and whether such divergence affects SCNT embryonic development, remains unclear. We thus compared both the global DNA hydroxymethylation and methylation dynamics between WT and SCNT embryos during pre‐implantation stages (**Figure** **5**A). Though 5hmC levels in gametes are initially lower than in somatic cells, the WT 2‐cell embryos exhibit higher hydroxymethylation than SCNT 2‐cell embryos, indicating more newly generated 5hmC during epigenetic reprogramming before the 2‐cell stage (Figure 5A). Meanwhile, with high DNA methylation levels in both parental alleles which are similar to sperm, SCNT genome displays hyper‐methylation before blastocyst stage. Besides that, the SCNT‐specific de novo methylation at 4–8 cell stage was also observed (Figure 5A). DhMRs were further identified between WT and SCNT embryos using published datasets^[^ ^24^ ^]^ (Figure 5B). We identified 4 categories of DhMRs with shared 5hmC level in both cell types during SZT: (1) SCNT‐specific regions, (2) WT‐specific regions, (3) commonly high‐5hmC regions, and (4) commonly low‐5hmC regions. Both SCNT‐ and WT‐specific DhMRs were identified as regions with 5hmC level difference >3% and q‐value <0.05, while stable regions were distinguished as regions with 5hmC difference <3% and q‐value <0.05.

The 5hmC accumulation in these four types of genomic regions soon decreased to comparable levels during 4–8 cell stage to the blastocyst stage before implantation (Figure 5B). Similarly, the DNA methylation level of these regions did not exhibit category‐specific variations different from the global methylation dynamics during pre‐implantation development (Figure S7A, Supporting Information). Notably, due to the significant global hyper‐methylation pattern in the SCNT genome before implantation, even the SCNT‐specific 5hmC regions displayed a higher 5mC level than the WT group at 2‐cell stage (Figure S7A, Supporting Information). These cell‐type‐specific DhMRs, together with stable high 5hmC regions, are preferentially enriched on the enhancers, as expected (Figure 5C; Figure S7B, Supporting Information). Furthermore, we assigned these enhancers to the target genes by proximity according to the published approach^[^ ^29^ ^]^ and subsequently screened for genes that were differentially expressed in WT and SCNT 2‐cell embryos with cell‐type‐specific DhMRs enriched on their enhancers, namely WT/SCNT‐specific genes (Figure 5D). Intriguingly, the WT‐specific genes were preferentially enriched in the physiological pathways related to early embryonic development identified by Gene Ontology (GO) analysis, such as cell fate commitment, pattern specification process, and regionalization etc. (Figure 5E). These results indicate that the aberrant 5hmC distribution on enhancers is closely associated with the developmental failure of SCNT embryos.

### 2.6 Parental‐Allele Specific 5hmC Dynamics in SCNT Embryos

**Figure 6:**
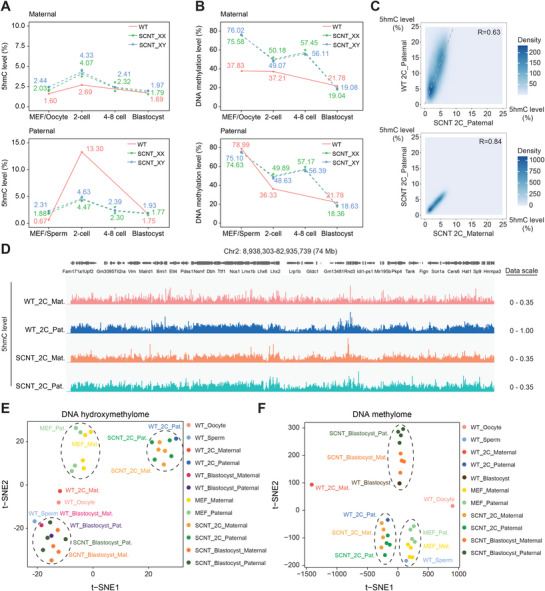
The 5hmC distribution on paternal and maternal genome from SCNT embryos compared with fertilized embryos. A,B) Dynamics of 5hmC (A) and DNA methylation (B) levels in both parental genomes during early embryonic development. The dynamics in WT embryos and SCNT embryos were represented by solid lines and dashed lines, respectively (*n* = 2 biological replicates for each gender at every stage, as shown in Table S1, Supporting Information). C) Density plots comparing 5hmC levels in 100‐kb tiles between WT paternal genome and SCNT paternal genome (top), and between SCNT parental genomes (bottom). The slope of the fitted line in the upper diagram is 1.98. D) Representative 5hmC level of maternal and paternal genomes in WT and SCNT 2‐cell embryos. E,F) 2D t‐distributed stochastic neighbor embedding (t‐SNE) visualization of DNA hydroxymethylation (E) and DNA methylation (F) profiles in 100‐kb tiles of the parental genomes in WT and SCNT embryos.

Although aberrant global DNA hyper‐methylation in SCNT 2‐cell embryos has been documented,^[^ ^15^ ^]^ the parental allele‐specific epigenetic differences between SCNT and WT embryos are yet to be elucidated. Allele‐specific comparison of 5hmC dynamics between SCNT and WT embryos revealed a striking divergence in parental genome asymmetry at 2‐cell stage, which gradually diminished and became virtually indistinguishable in the blastocysts. Specifically, at the 2‐cell stage, WT embryos exhibited significantly higher 5hmC levels on the paternal genome (13.3%) versus the maternal genome (2.69%). In contrast, SCNT embryos displayed comparable 5hmC distribution on both parental genomes (4.33% vs 4.63% in males, 4.07% vs 4.47% in females), leading to relatively insufficient 5hmC distribution in the paternal genome of SCNT 2‐cell embryos (**Figure** **6**A). Correspondingly, both parental genomes of SCNT embryos exhibited parallel DNA methylation dynamics across all pre‐implantation stages. Compared to the WT embryos, both parental genomes of SCNT embryos showed hypermethylation at the 2‐cell stage but matched the methylation level of WT embryos later at the blastocyst stage (Figure 6B). Nearly‐symmetric dynamics of both global 5hmC level and DNA methylation level on parental alleles of SCNT embryos motivated us to further investigate whether their genomic distribution patterns displayed such symmetry likewise.

In SCNT 2‐cell embryos, the 5hmC levels across 100‐kb genomic tiles are almost identical between the paternal and maternal genomes, showing a positive linear correlation (*k* = 1.98) with the 5hmC levels on the paternal genome of WT embryos (Figure 6C). Upon normalizing data scales across WT and SCNT samples, we were surprised to observe that both parental genomes in SCNT embryos exhibited 5hmC distributions resembling the WT paternal genome with overall lower levels, evident in both the chromosomal landscape (Figure 6D) and specific genomic sites (Figure S8, Supporting Information). However, such symmetric 5hmC distribution between parental genomes does not occur in WT 2‐cell embryos. Furthermore, the 5hmC profile of the maternal genome in SCNT 2‐cell embryos significantly differ from that in the WT 2‐cell maternal genome (Figure S9A, Supporting Information), indicating loss of WT maternal 5hmC pattern in SCNT 2‐cell embryos. Quantitative analysis of 5hmC levels at various developmental stages in both WT and SCNT embryos demonstrates that SCNT embryos reestablished a WT paternal‐like, allele symmetric 5hmC distribution during SZT reprogramming, which distinctly contrasts with the asymmetrical, paternal‐specific hyper‐hydroxymethylation seen in WT 2‐cell embryos. These 5hmC distribution abnormalities in SCNT embryos are gradually rescued at the blastocyst stage, as the global 5hmC decreases with the development progresses, resulting in a hydroxymethylation pattern similar to that of WT embryos (Figure 6E; Figure S9B, Supporting Information).

Correspondingly, the SCNT 2‐cell embryos displayed highly symmetrical DNA methylation distribution between parental genomes, mirroring the pattern in the donor MEF nuclei, albeit globally higher DNA methylation level (Figures S10 and S11A, Supporting Information). Such pattern is also distinct from the parental‐asymmetric DNA methylation profile observed in mouse gametes or WT 2‐cell embryos with higher 5mC level on paternal genome compared to maternal genome (Figure S11B, Supporting Information). Since the DNA methylation levels in the parental genomes of SCNT 2‐cell embryos do not resemble the distribution patterns of either WT paternal or maternal genomes (Figure S11C, Supporting Information), we turned to analyze the changes in DNA methylation level during the epigenetic reprogramming from somatic cell/germ cell to 2‐cell embryo. Surprisingly, we identified a strong linear correlation between the methylation level changes in SCNT genome (from MEF to SCNT 2‐cell stage) and those in WT paternal genomes (from sperm to 2‐cell stage) across the 10‐kb genomic tiles (Figure S11D, Supporting Information). However, the extent of DNA demethylation in SCNT genomes was generally lower. These results indicate that both parental genomes in SCNT mouse embryos undergo DNA demethylation in a sperm‐like profile during SZT, though at a lower level than the sperm, accompanied by the loss of WT maternal methylation pattern at the 2‐cell stage. In line with the parental allele‐specific 5hmC dynamic, the distinction of DNA methylation pattern between SCNT and WT embryos arose at the 2‐cell stage after the epigenetic reprogramming during SZT and subsequently diminished in blastocysts (Figure 6F; Figure S11E, Supporting Information), suggesting the presence of mechanisms that potentially correct the aberrant epigenome during mouse SCNT embryonic development. Collectively, our findings demonstrate that the oocyte cytoplasm mediates sperm‐like, though attenuated, epigenetic reprogramming of both parental genomes in the transferred somatic nuclei, resulting in transient allele‐symmetric DNA methylation and hydroxymethylation patterns in SCNT 2‐cell embryos that differ from those in WT embryos. These aberrations are resolved during development, with SCNT blastocysts ultimately exhibiting epigenetic profiles similar to those of WT blastocysts.

### 2.7 Excessive Level of 5hmC Disrupts the Epigenetic Landscape of SCNT Embryos

**Figure 7:**
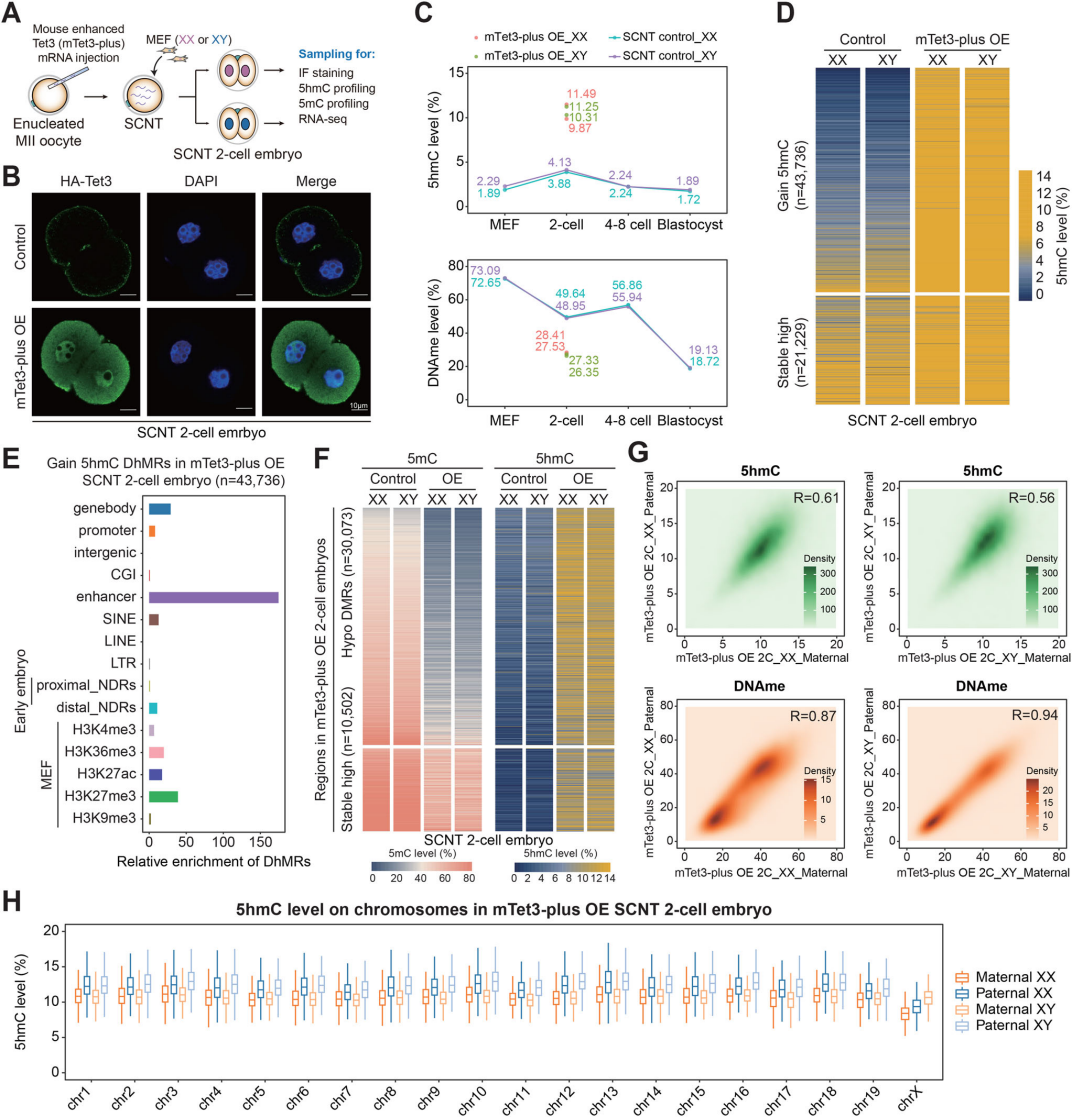
The 5hmC level and distribution in Tet3‐overexpressed SCNT embryos. A) Schematic illustration of the experimental approach. B) Immunostaining of HA in SCNT embryos injected with HA‐mTet3‐plus at 2‐cell stage. C) Dot plots showing 5hmCpG (top) and DNA methylation levels (bottom) in mTet3‐plus OE SCNT 2‐cell embryos, comparing with the dynamics in normal SCNT embryos (*n* = 2 biological replicates for each gender at every stage, as shown in Table S1, Supporting Information). D) Heatmaps showing 5hmCpG levels of both gain 5hmC DhMRs and stable high 5hmC regions in mTet3‐plus OE embryos compared to normal SCNT embryos at 2‐cell stage. E) Bar plots showing enrichment scores of gain 5hmC DhMRs in mTet3‐plus OE SCNT 2‐cell embryos across different genomic elements. F) Heatmaps showing 5mC and 5hmC levels at hypo DMRs and stable high methylated regions in SCNT 2‐cell embryos overexpressing mTet3‐plus compared to the control group. G) Density plots comparing 5hmC (top) and DNA methylation levels (bottom) in 100‐kb tiles between the paternal and maternal genomes in SCNT 2‐cell embryos overexpressing mTet3‐plus. H) Box plots showing 5hmC levels of 19 autosomes and the X chromosome in the paternal and maternal genomes from mTet3‐plus OE SCNT 2‐cell embryos of both genders (*n* = 2 biological replicates for each gender).

To further clarify the role of 5hmC in somatic‐to‐zygotic reprogramming during the SCNT embryogenesis, we employed a gain‐of‐function validation using a mouse Tet3 mutant (mTet3‐plus) with enhanced dioxygenase activity (**Figure** **7**A). The expression of mTet3‐plus was verified by immunofluorescence (Figure 7B). Significant elevation of DNA hydroxymethylation level with a sharp decrease of methylation level was observed in SCNT 2‐cell embryos overexpressed with mTet3‐plus (Figure 7C; Figure S12A, Supporting Information). Additionally, an increase of the proportion of high 5hmCpGs, together with the increase of 5hmC level on both parental genomes, was also shown in the mTet3‐plus overexpressing embryos of both genders (Figure S12B–E, Supporting Information), suggesting successful induction of excessive 5hmC generation. A total of 43736 and 21229 loci across the genome exhibited 5hmC generation and maintained stable 5hmC levels, respectively (Figure 7D). The loci exhibiting 5hmC generation or maintaining high 5hmC levels were predominantly enriched in enhancer regions, followed by the gene bodies and the regions modified with H3K27me3, H3K36me3, or H3K27ac in MEFs (Figure 7E; Figure S13A, Supporting Information). In contrast, the loci with stably low 5hmC levels that resist the function of mTet3‐plus were mainly enriched on the promoters (Figure S13B, Supporting Information). Not surprisingly, regions undergoing DNA demethylation showed a significant upregulation of 5hmC level in the embryo overexpressing mTet3‐plus (Figure 7F; Figure S14A,B, Supporting Information), confirming that the decreased 5mC level and increased 5hmC level induced by mTet3‐plus overexpression are closely correlated. Similar parental‐symmetric patterns of both DNA methylome and hydroxylmethylome were conserved in the SCNT 2‐cell embryos with an elevated level of 5hmC (Figure 7G; Figure S15A,B, Supporting Information). By comparing the 5hmC level of both autosomes and X chromosomes in parental genomes from mTet3‐plus overexpressed mouse embryos of both genders, we revealed that the 5hmC accumulation on X chromosomes of female embryos is still less than 5hmC distributed on autosomes or X chromosomes from male Tet3‐plus OE samples (Figure 7H; Figure S16A,B, Supporting Information). These results suggested that an excessive level of 5hmC induced by mTet3‐plus overexpression led to global hyper 5hmC generation is closely associated with more extensive DNA demethylation compared to SCNT 2‐cell embryos.

### 2.8 Ectopic Generation of 5hmC Pre‐Activates Genes and Impedes Developmental Potential in SCNT Embryos

**Figure 8:**
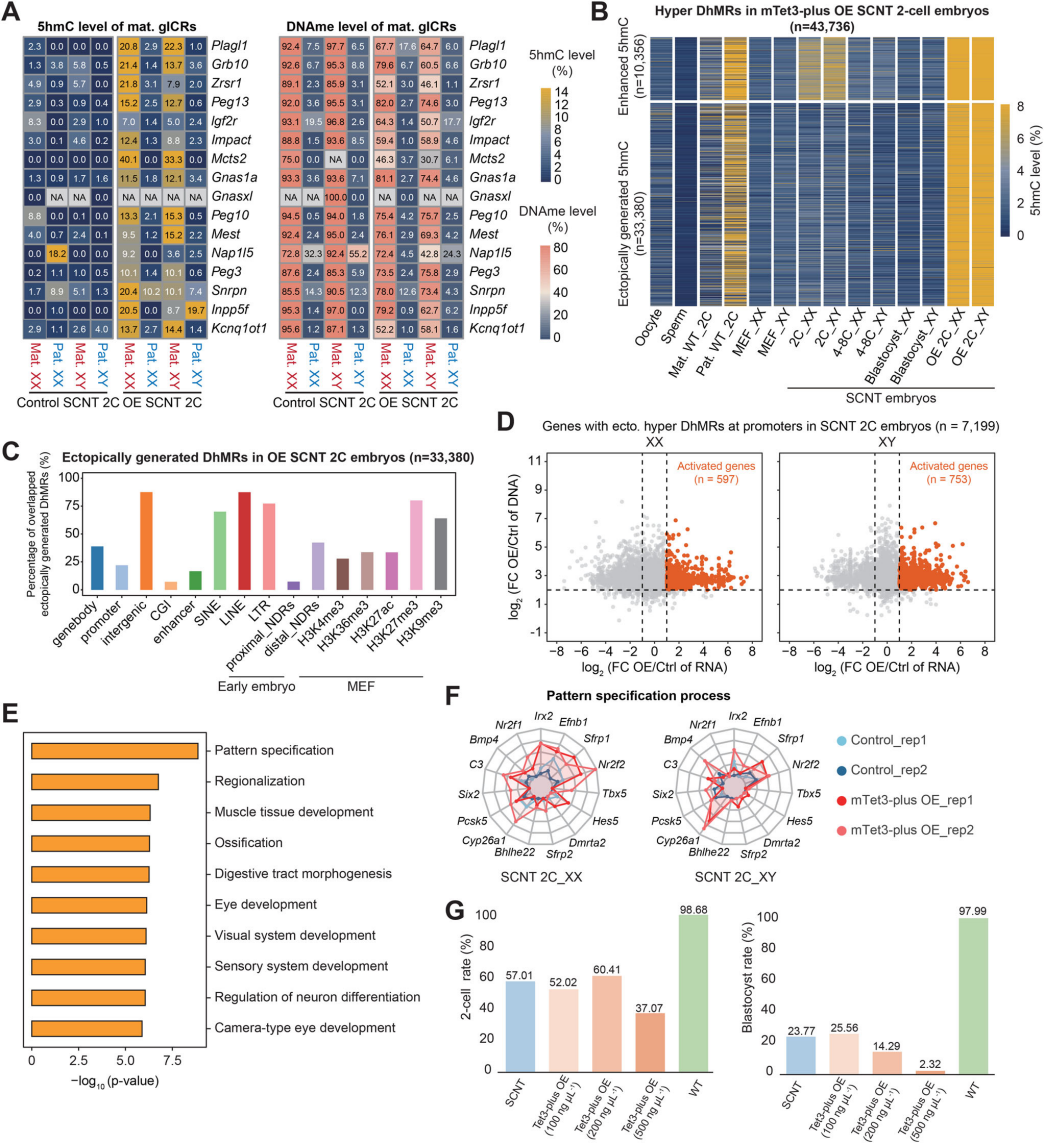
Schematic overview showing characteristics of 5hmC dynamics and distribution in SCNT embryos. A) Heatmaps showing 5hmC (left) and DNA methylation (right) levels at M_gICRs in both parental genomes from SCNT 2‐cell embryos overexpressing mTet3‐plus compared with the control SCNT 2‐cell embryos (*n* = 2 biological replicates). B) Heatmap showing the dynamics of 5hmCpG levels on hyper DhMRs of embryos during early embryo development. Enhanced DhMRs and ectopically generated DhMRs were defined based on its original 5hmC level in the control SCNT 2‐cell embryos. C) Bar plots showing the percentage of ectopically generated hyper‐DhMRs overlapping with various genomic elements. D) Scatter plots comparing gene expression level changes (*x*‐axis) and 5hmC/5mC level changes (*y*‐axis) between mTet3‐plus OE and control SCNT 2‐cell embryos. Dots represent genes with 5hmC ectopically generated at promoter regions. Potential reactivated genes caused by excessive 5hmC generation were colored orange. E) Bar plot showing Gene Ontology (GO) enrichment analysis of genes reactivated in mTet3‐plus OE SCNT 2‐cell embryos identified in Figure 8D. F) Radar chart demonstrating the expression levels of genes associated with selected Gene Ontology (GO) terms in Figure 8E. G) Bar plots showing the proportion of embryos injected with mTet3‐plus mRNA that developed to the 2‐cell stage (left) and blastocyst stage (right) (For SCNT group, sample size *n* = 214; Tet3‐plus OE 100 ng µL^−1^ group, *n* = 173; Tet3‐plus OE 200 ng µL^−1^ group, *n* = 197; Tet3‐plus OE 500 ng µL^−1^ group, *n* = 116; WT group, *n* = 151).

To further explore the impact of extensive DNA demethylation and excessive 5hmC generation on SCNT embryonic development, we first examined the DNA hydroxymethylation and methylation level on genomic imprinting regions in the genome of mTet3‐plus overexpressed embryos. Unlike the SCNT embryos where maternal gICRs can resist the first wave of demethylation at 2‐cell stage and undergo imprinting loss during the second wave of demethylation, the 15 maternal gICRs from the genome of mTet3‐plus overexpressed SCNT embryos experienced elevated 5hmC levels and varying degrees of imprinting loss during the first wave of demethylation (**Figure** **8**A). A total of 43 736 loci were identified as hyper DhMRs in mTet3‐overexpressed embryos (Figure 8B). Among these, 10 356 DhMRs originally exhibited high 5hmC levels in SCNT 2‐cell embryos, with the level being further enhanced in the OE group. The remaining 33 380 DhMRs experienced ectopic 5hmC generation that normally do not undergo significant 5hmC elevation during the SCNT 2‐cell stage (Figure 8B). And 7199 of these ectopically generated DhMRs were overlapped with the promoter regions of genes, and 5438 of them were overlapped with enhancer regions, suggesting potential influence on gene expression in SCNT embryos (Figure 8C).

To identify genes affected by ectopically generated 5hmC, we screened for the gene candidates based on the 2 criteria: (1) Transcriptional reactivation: low expression or silence in SCNT 2‐cell embryos but activated in mTet3‐plus overexpressed embryos; (2) Enrichment of ectopically generated DhMRs coupled with significant DNA demethylation. This approach yielded 597 aberrantly reactivated genes in female mTet3‐OE embryos and 753 in males with ectopically generated 5hmC on promoters (Figure 8D), before subsequently subjected to GO analysis to elucidate their functional enrichments (Figure 8E). Intriguingly, the genes reactivated at 2‐cell stage by excessive 5hmC were mainly enriched in pathways such as “pattern specification,” “regionalization,” or “ossification,” which are developmental processes expected to occur during or after gastrulation in SCNT embryos (Figure 8F). Analysis of reactivated genes with ectopically generated 5hmC on the enhancers using the same workflow yielded a consistent conclusion (Figure S17A–D, Supporting Information). Correspondingly, we observed that the two‐cell rate and blastocyst rate of SCNT embryos overexpressing mTet3‐plus were lower than those of the control group, independent of the dosage applied (Figure 8G), with abnormal embryonic morphology (Figure S18, Supporting Information). These results implied that ectopically generation of 5hmC induced by mTet3‐plus pre‐activates genes involved in subsequent developmental events at 2‐cell stage, leading to disruptions and failure of SCNT development.

## 3 Discussion

Our study provides the first comprehensive analysis of 5hmC dynamics during somatic cell nuclear reprogramming, revealing distinct differences between SCNT and fertilized embryos while identifying DNA hydroxymethylation defects as a critical epigenetic barrier in mammal cloning. We found that both parental genomes in SCNT embryos adopt an attenuated, sperm‐like 5hmC profile, suggesting the ooplasm drives symmetric paternal‐like DNA hydroxymethylation regardless of genomic origin. This contrasts sharply with normal fertilization, where 5hmC is predominantly generated on the paternal genome,^[^ ^24^ ^]^ highlighting different epigenetic reprogramming dynamics in SCNT.

The tight linkage between 5hmC generation and DNA demethylation specifically during SZT suggests TET3‐mediated oxidation may facilitate initial epigenetic reprogramming in cloned embryos, analogous to its role in paternal genome demethylation after fertilization.^[^ ^22^ ^]^ However, the subsequent uncoupling of 5hmC and methylation dynamics indicates this mechanism becomes largely abolished during later development. Notably, the symmetric 5hmC patterns in SCNT embryos coincide with loss of maternal‐specific epigenetic signatures, potentially reflecting competition between oocyte reprogramming factors and somatic memory. This may explain why SCNT embryos require additional epigenetic modifiers like HDAC inhibitors to achieve better reprogramming.^[^ ^30^ ^]^

The X chromosome represents insufficient 5hmC accumulation persists despite global hydroxymethylation elevation in SCNT embryos. This X‐specific resistance may reflect inherent chromatin features that limit TET3 access or resistant to demethylation mechanisms. As X chromosome dosage compensation is critical for development,^[^ ^31^ ^]^ this deficiency could contribute to the aberrant Xist expression and premature X inactivation in SCNT embryos. Interestingly, even TET3 overexpression failed to normalize X chromosome hydroxymethylation in SCNT embryos, suggesting additional regulation factors may exist beyond TET3‐mediated oxidation.

Allele‐specific analyses revealed that gICRs escape the initial wave of 5hmC‐mediated demethylation, only to undergo 5hmC‐independent methylation loss later. These findings may reflect distinct mechanisms protecting and erasing imprints during development. The eventual gICR demethylation likely contributes to the imprinting disorders observed in cloned animals.^[^ ^32^ ^]^ Notably, TET3 overexpression induced premature gICR demethylation coupled with 5hmC accumulation, demonstrating that excessive hydroxymethylation can destabilize these normally protected regions. This suggests tight regulation of 5hmC levels is crucial for maintaining genomic imprints. Moreover, we found that excessive 5hmC by TET3 overexpression in SCNT embryos caused severe developmental arrest. The premature activation of late developmental genes at the 2‐cell stage, especially patterning and ossification factors, suggests 5hmC misregulation disrupts the precise temporal coordination of embryonic gene expression programs.

A particularly intriguing aspect of our findings is the distinct 5hmC dynamics in SCNT embryos compared with fertilized counterparts. One notable feature is the insufficient generation of 5hmC on the X chromosome—especially the maternal X—during the stage of zygotic transcription (SZT). In fertilized embryos, TET3 targeting is tightly linked to genomic context, including DNA methylation, histone modifications, and chromatin accessibility. The deficiency observed in SCNT embryos may therefore reflect particularly strong barriers on the X chromosome that resist DNA hydroxymethylation. In parallel, SCNT embryos exhibit allele‐symmetric DNA hydroxymethylation and demethylation across parental genomes, in contrast to the asymmetric patterns typically seen in wild‐type embryos. This aberrant symmetry likely reflects the nuclear donor's epigenetic legacy, imposing a homogenized state that masks normal paternal–maternal asymmetry and may disrupt proper lineage‐specific gene regulation.

Despite these abnormalities, the SCNT‐specific 5hmC landscape is not static. We observed a gradual recovery of 5hmC distribution from the 2‐cell stage toward the blastocyst stage, potentially facilitated by compensatory mechanisms such as delayed recruitment of TET1/2, global DNA demethylation, or chromatin remodeling events that progressively reset the donor genome toward a more embryonic‐like state. Together, these observations support a model in which SCNT embryos undergo an initial phase of impaired and homogenized hydroxymethylation, followed by a progressive but incomplete recovery of epigenetic asymmetry. Validating the underlying mechanisms, however, would require precise locus‐specific reprogramming technologies capable of rescuing 5hmC distribution specifically on the X chromosome or paternal genomes. Such approaches remain challenging due to current limitations in efficiency and off‐target effects. This represents a key limitation of our study, but future advances in these technologies could be expected to provide strategies to correct 5hmC aberrations and further improve the developmental competence of SCNT embryos.

The normalization of 5hmC patterns by the blastocyst stage suggests compensatory mechanisms eventually correct some hydroxymethylation defects. This may involve passive dilution through cell divisions or active remodeling during embryonic genome activation. However, given the low viability of SCNT embryos, this rescue appears insufficient for normal development. While the oocyte cytoplasm can partially reprogram somatic nuclei, its inability to establish proper hydroxymethylation asymmetry appears limiting. Our findings suggest future efforts to optimize SCNT should achieve more precise spatiotemporal control of 5hmC deposition during nuclear reprogramming.

Lastly, although this study provides a comprehensive genome‐wide view of DNA hydroxymethylation and methylation dynamics in SCNT embryos, several limitations should be acknowledged. Our analyses were based on low‐input ACE‐seq and WGBS datasets generated from ≈200 cells per sample, rather than at the single‐cell level. Consequently, cell‐to‐cell heterogeneity could not be fully resolved, and subtle epigenetic variations among individual blastomeres may have been overlooked. In addition, our data cannot directly address the developmental potential or reprogramming competence of individual SCNT embryos. Future studies employing single‐cell multi‐omics approaches and embryo transfer experiments will be required to further elucidate the relationship between epigenetic remodeling and developmental competence in nuclear transfer embryos.

## 4 Experimental Section

### Animal Maintenance

WT C57BL/6J mice were purchased from Vital River (China) and the Animal Center of the Institute of Zoology, Chinese Academy of Sciences. The PWK/PhJ strain mice were purchased from Jackson Laboratory (USA). B6D2F1 hybrid mice (C57BL/6×DBA/2) were housed and bred in a specific pathogen‐free (SPF) facility with controlled temperature and a 12‐h light/12‐h dark cycle, and provided with water and standard chow ad libitum. All the maintenance and experimental procedures of animals were reviewed and approved by the Institutional Animal Care and Use Committee of the Institute of Zoology of the Chinese Academy of Sciences (IOZIACUC2020‐107).

### Statement on Exclusion of Samples and Data

No statistical methods were employed to predetermine sample size. For all experiments described in this study, mouse MII oocytes and MEFs were collected and randomly assigned to control or treatment groups without prior selection based on morphology or cellular condition. Data collection and analysis were conducted with full knowledge of the experimental groups, without blinding. No MEFs, SCNT early embryos, or individual data points were excluded from analysis for any reason.

### Preparation of MEFs as Donor Nuclei for Somatic Cell Nuclear Transfer

Mouse embryonic fibroblasts (MEFs) derived from E13.5 embryos were used as nuclear donors in the Somatic Cell Nuclear Transfer (SCNT) experiments. These embryos were obtained by mating C57BL/6J female mice with PWK/PhJ male mice. At embryonic day 13.5 (E13.5), pregnant females were euthanized, and embryos were harvested under a stereomicroscope. The head, tail, limbs, internal organs, and visible blood vessels were carefully removed, retaining only the dorsal body tissue for further processing. Sex determination of each embryo was initially assessed based on the morphology of the genital ridge and subsequently confirmed by PCR genotyping targeting the *Sry* gene. Embryonic tissues were grouped based on sex and transferred into new 10‐cm culture dishes for dissociation. Each tissue sample was minced for 5 min, then digested by adding 6 mL of 1× TrypLE Express (Gibco, Cat#12604‐021). The tissues were incubated at 37 °C for 15 min, with gentle shaking every 5 min to enhance enzymatic digestion. To stop the digestion, 8 mL of MEF culture medium (Dulbecco's Modified Eagle's Medium (DMEM) supplemented with 10% fetal bovine serum (Vistech, Cat#SE100‐011) and 0.5% penicillin–streptomycin (Gibco, Cat#15140122) was added. The cell suspension was then transferred to 15‐mL centrifuge tubes and centrifuged at 300 ×g for 5 min. The supernatant was discarded, and the cell pellet was resuspended in fresh MEF medium. Cells were counted and plated in 10‐cm culture dishes to obtain passage 0 (P0) MEFs. Cultures were maintained in a humidified incubator at 37 °C with 5% CO_2_. After 12 h, the medium was removed, and cells were washed twice with 1× DPBS. Fresh MEF medium (10 mL) was added for continued culture. Cells were passaged at a ratio of 1:3 when they reached ≈80% confluence, typically 2–3 days later, until passage 3 (P3) was achieved. Before cryopreservation, P3 MEFs were tested for mycoplasma contamination using PCR, and confirmed that they were negative for mycoplasma contamination. P3 MEFs were then harvested and cryopreserved at a density of 1 × 10⁶ cells per cryovial in freezing medium composed of DMSO: FBS: MEF medium at a ratio of 1: 4: 5. Vials were stored in liquid nitrogen until use for nuclear transfer experiments.

### Somatic Cell Nuclear Transfer Using MEF Donor Nuclei

Mature metaphase II (MII) oocytes were collected from super‐ovulated B6D2F1 females (8–10 weeks), 14–16 h post‐human chorionic gonadotropin (hCG) injection. Cumulus cells were removed using 0.3 mg mL^−1^ hyaluronidase (Sigma, Cat#H3884) in homemade HEPES‐CZB medium. Oocytes were then transferred to manipulation HEPES‐CZB medium containing 5 µg mL^−1^ cytochalasin B (CB, Sigma, Cat#C6762) and enucleated under a micromanipulation system using a Piezo‐driven pipette. After enucleation, the spindle‐free oocytes were washed extensively and maintained in homemade CZB medium up to 1 h before nucleus injection. Thawed P3 MEF donor cells were briefly cultured and synchronized in the G0/G1 phase of the cell cycle by culture in medium with low serum (DMEM/F12 medium with 0.5% FBS) for 2–4 days before SCNT. Then, individual donor cells were injected into the perivitelline space of enucleated oocytes using a Piezo‐driven microinjection pipette. The reconstructed oocytes were cultured in CZB medium for 1 h and then activated for 6 h in activation medium containing 10 mM Sr^2+^ (Sigma, Cat#S0390), 5 ng mL^−1^ trichostatin A (TSA, Sigma, Cat#T8552), and 5 µg mL^−1^ CB at 37 °C. Following activation, all of the reconstructed embryos were cultured in KSOM medium (Millipore, Cat#MR‐106‐D) supplemented with 5 ng mL^−1^ TSA for another 4 h and maintained in KSOM medium under mineral oil at 37 °C in a 5% CO_2_ incubator. The SCNT 2‐cell embryos, 4 to 8‐cell embryos, and blastocysts were collected at 24–26 h, 48–50 h, and 96–100 h post‐injection, respectively. For ACE‐seq and WGBS, the average numbers of SCNT 2‐cell, 4 to 8‐cell embryos, and blastocysts that were pooled as one biological replicate were 100, 50, and 20, respectively. Two biological replicates were performed for each sample, which represents the accepted standards in studies of early embryonic epigenomes. For RNA‐seq, the average number of SCNT 2‐cell embryos that were pooled as one biological replicate was 7; two biological replicates were included for each group. All attempts at replication were successful.

### Construction of the ACE‐Seq Libraries

ACE‐seq libraries were constructed following previously established protocols.^[^ ^24^ ^]^ For E13.5 MEFs, genomic DNA (gDNA) was extracted by using DNeasy Blood & Tissue Kits (Qiagen, Cat#69504) according to the manufacturer's protocol, and 200 ng of gDNA was then subjected to the ACE‐seq protocol. For SCNT embryos, they were collected into 20 µL of RLT Plus buffer (Qiagen, Cat#1053393), briefly vortexed, and incubated at room temperature for 10 min to release gDNA. The gDNA was then purified and fragmented to an average size of ≈200 bp using a Covaris ultrasonicator. The sheared DNA was purified once using AMPure XP beads (Beckman Coulter, Cat#A63882). The purified gDNA was subjected to glucosylation by incubation with UDP‐glucose and T4 Phage β‐glucosyltransferase (New England Biolabs, Cat#A63882) at 37 °C for 1 h. Following glycosylation, the DNA was purified again with AMPure XP beads to remove residual enzymes and unincorporated nucleotides. Next, the DNA was denatured by adding 0.1 M NaOH and incubating at 55 °C for 10 min. Deamination was carried out using the APOBEC enzyme (New England Biolabs, Cat#E7125L) at 37 °C for 3 h. After deamination, the DNA was purified once more with AMPure XP beads before proceeding to library construction. Libraries were generated using the TAILS method^[^ ^33^, ^34^ ^]^ (Tailing and Ligation‐Free Method for Single Cells). The resulting ACE‐seq libraries were sequenced on an Illumina NovaSeq 6000 platform using 150‐bp paired‐end reads.

### Construction of the WGBS Libraries

As outlined in the ACE‐seq protocol, genomic DNA (gDNA) from both E13.5 MEFs and SCNT embryos underwent bisulfite conversion and subsequent purification using the EZ‐96 DNA Methylation‐Direct MagPrep Kit (Zymo Research, Cat#D5044), following the manufacturer's instructions. Whole‐genome bisulfite sequencing (WGBS) libraries were then prepared using the recently developed TAILS method. Briefly, the first round of random priming was carried out with the primer P5‐N6‐oligo1 (5′‐CTACACGACG‐CTCTTCCGATCTN6‐3′) in the presence of the Klenow exo(–) fragment (Qiagen). Unincorporated primers and nucleotides were subsequently removed using Exo‐SAP IT Express (Applied Biosystems, Cat#75001). A dC tailing reaction was then performed using terminal deoxynucleotidyl transferase (TdT; Thermo Fisher, Cat#EP0162). The second round of priming was conducted using P7‐G6‐oligo2 (5′‐AGACGTGTGCTCTTCCGATCTG6‐HN‐3′) in the presence of Klenow exo(–). The resulting products were purified with AMPure XP beads (Beckman Coulter, Cat#A63882), followed by PCR amplification to generate the final libraries. After a final purification step, the libraries were assessed for size distribution using a Fragment Analyzer. Finally, the WGBS libraries were sequenced on an Illumina NovaSeq 6000 platform using 150 bp paired‐end sequencing.

### Construction of RNA‐Seq Libraries

RNA‐seq libraries were generated using the Smart‐seq2 protocol. ≈7 mouse SCNT embryos were collected per biological replicate. Briefly, embryos were manually picked and transferred into the lysis buffer. Polyadenylated (poly(A)^+^) RNA was captured using oligo(dT) primers. First‐strand cDNA synthesis was performed via reverse transcription, followed by amplification to generate full‐length cDNA. The amplified cDNA was subsequently purified and fragmented. The resulting cDNA fragments were used to construct RNA‐seq libraries with the NEBNext Ultra II DNA Library Prep Kit (New England Biolabs, Cat#E7645L), following the manufacturer's instructions. Final libraries were sequenced on an Illumina NovaSeq 6000 platform using a 150 bp paired‐end read configuration.

### In Vitro Transcription and Microinjection

The mouse enhanced Tet3 (mTet3‐plus) plasmid was constructed as previously described.^[^ ^28^ ^]^ To prepare the mTet3‐plus mRNA, the plasmid was linearized, and capped mRNA was synthesized using the EasyCap T7 Co‐transcription Kit with CAG Trimer (Vazyme, Cat#DD4203), following the manufacturer's protocol. The transcribed mRNA was then purified using VAHTS RNA Clean Beads (Vazyme, Cat#N412‐01) and eluted in nuclease‐free water. RNA integrity was verified using the 5200 Fragment Analyzer System (Agilent) to ensure high‐quality transcripts. For microinjection, ≈10 pL of mTet3‐plus mRNA (100, 200, or 500 ng µL^−1^) was injected into enucleated MII oocytes using a FemtoJet microinjector (Eppendorf) under a constant flow setting. Following mRNA injection, SCNT was performed. The injected cloned embryos were cultured in vitro. At the 2‐cell stage, embryos were collected for downstream epigenomic analyses, including ACE‐seq and WGBS with detailed sequencing metrics summarized in Table S1 (Supporting Information). In parallel, 2‐cell embryos were harvested for transcriptome profiling using the Smart‐seq2 assay, with data statistics provided in Table S2 (Supporting Information). In parallel, immunostaining was performed to detect HA‐mTet3‐plus protein, as well as the levels of 5‐hydroxymethylcytosine (5hmC) and 5‐methylcytosine (5mC).

### Immunofluorescence Staining of Mouse 2‐Cell Embryos

Immunofluorescence detection of proteins in mouse 2‐cell embryos was performed following the previously published procedures.^[^ ^24^ ^]^ Briefly, the zona pellucida was first removed with Tyrode's solution, and the control and SCNT 2‐cell embryos were subsequently fixed with 3.7% PFA for 1 h. The embryos were permeabilized with 0.2% Triton X‐100 for 30 min and blocked in 3% BSA for 1 h at room temperature (RT). Then, the embryos were incubated with primary antibodies at 4 °C overnight. After washing with 0.05% Tween‐20, the embryos were incubated with secondary antibodies for 1.5 h at RT. Finally, the embryos were mounted and transferred to a confocal dish. All immunofluorescence images were captured using a Zeiss LSM‐880 confocal microscope. The primary antibodies used in this study were as follows: Anti‐HA (Abcam, Cat#ab18181); Anti‐5hmC (Active motif, Cat#39791); Anti‐5mC (Diagenode, Cat#C15200081‐100). The second antibodies used in this study were as follows: Goat anti‐Mouse Alexa Fluor 488 (Invitrogen, Cat#A11029); Goat anti‐Rabbit Alexa Fluor 568 (Invitrogen, Cat#A‐11036).

### Processing of RNA‐Seq Data

For bulk RNA data analysis, raw sequencing reads were processed using the following pipeline. First, quality control and adapter trimming were performed using Trim Galore (v0.6.7). The trimmed reads were then aligned to the reference genome using STAR (v2.7.9a) with default parameters. The resulting BAM files were sorted and indexed using SAMtools (v1.14). Finally, gene expression quantification was performed using featureCounts (v2.0.6) with the following parameters: paired‐end mode, counting read pairs, and using exon features with gene_id as the identifier. To identify differentially expressed genes (DEGs) between SCNT and Tet3 OE embryos at the 2‐cell stage, the DESeq2 package (v1.34.0) in R was used. A count matrix of raw read counts was input into DESeqDataSetFromMatrix(). Three biological replicates were included per condition. Normalization was performed using DESeq2's internal method, and log2(normCounts+1) were exported for downstream visualization. DEGs were identified using the results function, applying the following thresholds: *p*‐value ≤ 0.01 and absolute log2 Fold Change ≥ 1 (corresponding to ≥two‐fold change). To ensure biological relevance, only genes with a minimum expression level of >1 normalized count in all samples were retained for DEG analysis. To investigate the biological functions associated with genes differentially expressed upon Tet3 overexpression, Gene Ontology (GO) enrichment analysis was performed using the clusterProfiler package (v4.2.1) in R. Separate analyses were conducted for upregulated and downregulated genes. Mouse gene symbols were used as input, and enrichment was performed against the org. Mm.eg.db annotation database with keyType = “SYMBOL.” The analysis focused on the Biological Process (BP) ontology, using the Benjamini–Hochberg method for multiple testing correction. GO terms with a q‐value ≤0.05 were considered significantly enriched.

### Processing of Whole Genome Bisulfite Sequencing (WGBS) Data

Raw sequencing reads were first subjected to quality control using Trim Galore (v0.6.10) to remove adapter sequences and low‐quality bases (Phred score < 20), and to discard reads shorter than 20 bp. Clean reads were then aligned to the mouse reference genome (mm9) using Bismark (v0.23.1) in paired‐end mode; unmapped reads were re‐aligned in single‐end mode. The resulting BAM files were processed with sambamba (v0.8.2) and samtools (v1.17) for duplicate removal and coordinate sorting. Cytosine methylation levels at CpG, CHH, and CHG (H = A, T, or C) were quantified using MethylDackel (v0.6.1).

### Processing of Whole‐Genome ACE‐Seq Data

Raw paired‐end ACE sequencing data were processed as previously reported. First, quality control and adapter trimming were performed using Trim Galore (v0.6.10). The processed reads were aligned to the reference genome mm9 using Bismark (v0.23.1). The alignment was performed in two steps: first, paired‐end alignment with unmapped reads output, followed by local alignment of the unmapped reads. The resulting BAM files were sorted and merged using sambamba (v0.8.2) and samtools (v1.17). Duplicate reads were removed using sambamba markdup. 5hmC levels were extracted using MethylDackel (v0.6.1) for CpG, CHG, and CHH contexts.

### Subtracting 5hmC from the WGBS Data by Combining the ACE‐Seq Dataset

A comprehensive comparison between ACE‐seq and WGBS data was performed. First, overlapping CpG sites between ACE‐seq and WGBS datasets were identified using bedtools intersect (v2.30.0). The methylation levels from both methods were then processed and formatted into bedGraph files. To accurately estimate the levels of 5mC, 5hmC, and unmodified cytosines at each CpG site, the MLML tool (v5.0.0) was used with ACE‐seq and WGBS data as inputs. CpG sites with negative levels or conflicts were filtered out from the downstream analysis.

### Identifying Allele‐Specific Alignments from Sequencing Data

To distinguish between maternal and paternal alleles, SNPsplit (v0.5.0) was employed in conjunction with the SNP database. Briefly, raw sequencing reads from C57BL/6J and PWK/PhJ mouse strains were trimmed and aligned as described above. Reads were mapped to an N‐masked mm9 reference genome, which used all known PWK/PhJ SNPs from the Mouse Genomes Project. Following alignment, reads were classified as maternal (C57BL/6J) or paternal (PWK/PhJ) based on SNP identity, enabling allele‐specific downstream analysis.

### Annotation of Genomic Regions

Genomic annotations, including CpG islands (CGIs), exons, introns, transcription start sites (TSSs), and transcription end sites (TESs), were obtained from the UCSC Genome Browser (mm9). Gene bodies were defined as the regions between TSSs and TESs, and intergenic regions were defined as the complementary genomic regions outside gene bodies. Promoters were defined as 1000 bp upstream and 500 bp downstream of TSSs. Nucleosome‐depleted regions (NDRs) in early mouse embryos, germline differentially methylated regions (gDMRs) of maternal and paternal genomes, and histone modification peaks were obtained from previously published datasets. Repetitive elements were annotated using RepeatMasker (mm9). Enhancer centers in the mm9 genome were derived from a previously published study.^[^ ^29^ ^]^ The assignment of enhancers to the related genes was conducted by proximity, which was previously described.^[^ ^24^ ^]^ Specifically, the enhancers were assigned to the nearest gene (distance to TSS  < 100  kb) using BETA (1.0.7).

### Identification of DMRs

Differentially methylated regions (DMRs) were identified based on replicate aggregation. The mouse genome (mm9) was divided into 100‐bp non‐overlapping windows, and only those containing at least two CpG sites were retained. A sliding window approach (1000 bp window size, 300 bp step size) was used to perform Student's *t*‐tests between groups. Overlapping windows with *p* < 0.1 were merged. To classify DMRs, CpG methylation levels were compared between two groups (Groups A and B). Windows with a mean methylation difference > 0.25 were defined as hypermethylated DMRs; those with differences <−0.25 were defined as hypomethylated DMRs. And regions with absolute differences < 0.15 and mean methylation levels > 0.65 in both groups were considered stable high DNA methylation regions. For hydroxymethylated DMRs (DhMRs), regions with absolute differences > 0.03 were classified as hyper or hypo DhMRs. Regions with absolute differences < 0.03 and mean 5hmC levels > 0.05 in both groups were considered stable high. Regions of the same class located within 10 kb were merged. Regions containing at least three CpG sites in the aggregated dataset and showing significant differences (false discovery rate–adjusted *p* < 0.1, multiple *t*‐tests) were retained as DMRs or DhMRs. Finally, loci with no significant differences (FDR‐adjusted *p* > 0.05) were classified as stable methylated regions.

### Enrichment of DMRs at Genomic Elements

For DMR enrichment, a Fisher's exact test was performed using the cumulative lengths of DMRs and background genome regions. Enrichment score was quantified as −log_10_ (*p*‐value).

### t‐SNE Analysis of the 5mCpG & 5hm CpG and DNA Methylation Level

The mouse genome (mm9) was partitioned into non‐overlapping 100‐kb tiles. For each tile, 5mCpG and 5hmCpG levels were quantified and compiled into a matrix. The resulting matrix was scaled using the scale function in R to standardize values across features. Dimensionality reduction was performed using t‐distributed stochastic neighbor embedding (t‐SNE) via the Rtsne package (parameters: dims = 2, perplexity = 2) to visualize sample relationships.

### Statistical Analysis

For box plots, the boxes indicate the interquartile range (IQR), with the lower and upper edges representing the 25th and 75th percentiles, respectively; the center line denotes the median. Whiskers extend to the most extreme data points within 1.5× IQR from the box edges. For bar plots, error bars represent the mean ± standard error of the mean (s.e.m.). Statistical significance was assessed using two‐sided Wilcoxon's rank‐sum test or Student's *t*‐test, as indicated. Significance thresholds were defined as: *p < 0.05; **p < 0.01; ***p < 0.001; ****p < 0.0001; NS, not significant. For genomic track visualizations, values were scaled to a range of 0–1 or 0–0.2, as specified.

## Conflict of Interest

The authors declare no conflict of interest.

## Author Contributions

Z.X., R.Y., J.G., and M.W. contributed equally to this work. F.G. and D.L. conceived and supervised the study. D.L. and M.W. performed SCNT embryo experiments. R.Y. conducted high‐throughput sequencing experiments. Z.X. analyzed and integrated the data. X.C. performed an immunofluorescence staining assay. F.Z. and T.G. did molecular cloning. J.G. and X.L. completed the bioinformatic analysis. Z.X., F.G., and D.L. wrote the paper with help from all the authors.

## Supporting information



Supporting Information

Supplemental Table 1

Supplemental Table 2

## Data Availability

The data that support the findings of this study are openly available in the National Genomics Data Center at https://bigd.big.ac.cn, reference number CRA025579 and OMIX010093.
