# Supplementary material for: 5‐Hydroxymethylcytosine Dynamics Reveals Coordinated Reprogramming of Parental Genomes and X Chromosome Dosage Balance in Mouse SCNT Embryos
Source: Adv Sci (Weinh). 2025 Nov 18;13(6):e09682. doi: 10.1002/advs.202509682 (PMC12866825; doi:10.1002/advs.202509682)
Supplement: Supplementary file 1 — Supporting Information [file ADVS-13-e09682-s001.pdf]

Supporting Information

5-hydroxymethylcytosine Dynamics Reveals Coordinated Reprogramming of Parental Genomes and X Chromosome Dosage Balance in Mouse SCNT Embryos

Zeming Xiang, Rui Yan, Jing Guo, Mengyao Wang, Xin Cheng, Fan Zhang, Tianzi Guo, Xin Long, Fan Guo[*]*, Dan Liang*[*]

The file includes Figure S1-S18.

Other Supporting Information for this manuscript includes Table S1-S2.

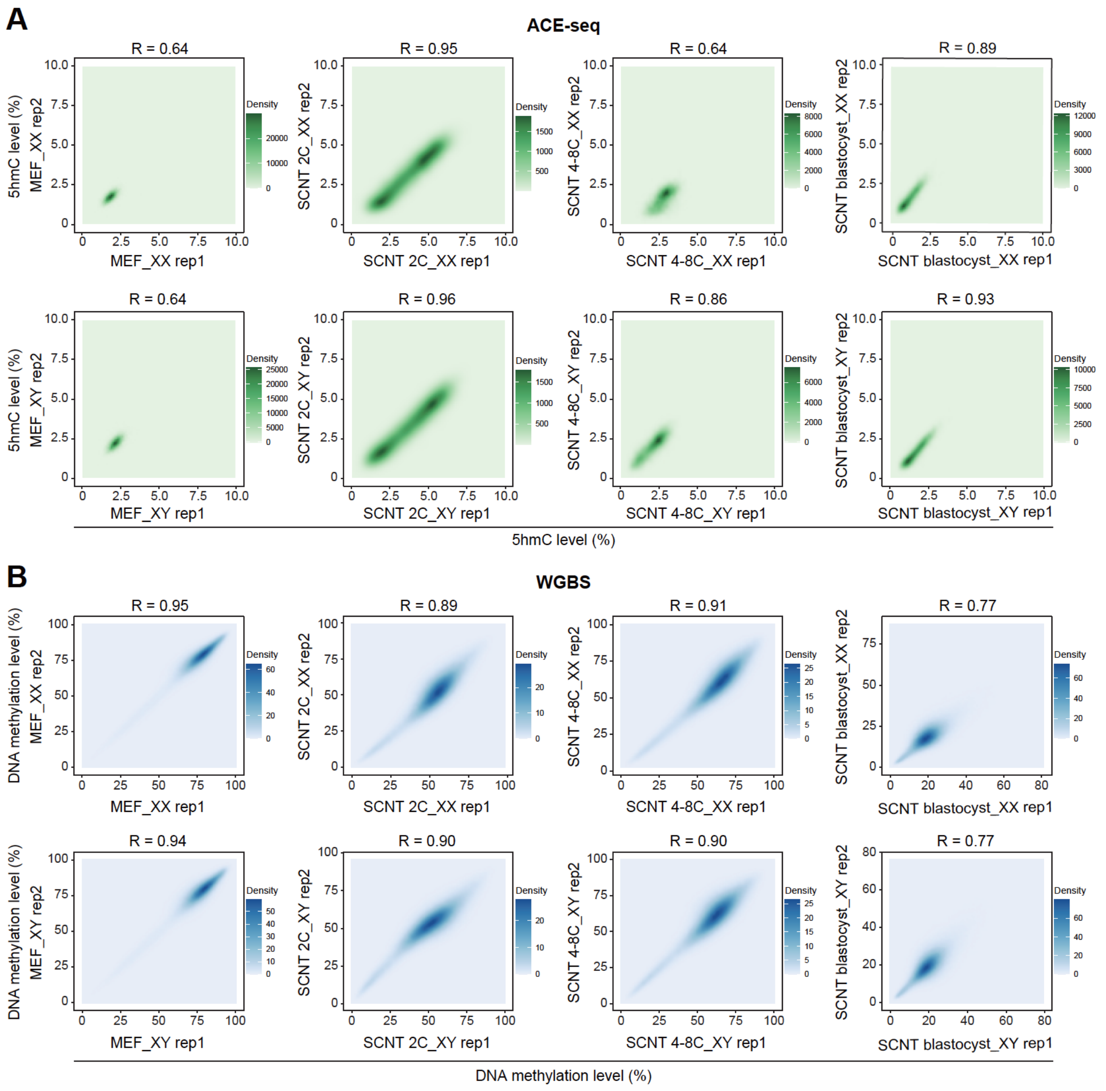

Figure S1. Quality assessment of 5mC & 5hmC sequencing data and 5hmC dynamics.

A-B, Density plots illustrating the 5hmC (A) and DNA methylation (B) levels between two biological replicates across genome tiles. The 5hmC data was acquired by ACE-seq, and the DNA methylation data was obtained by WGBS.

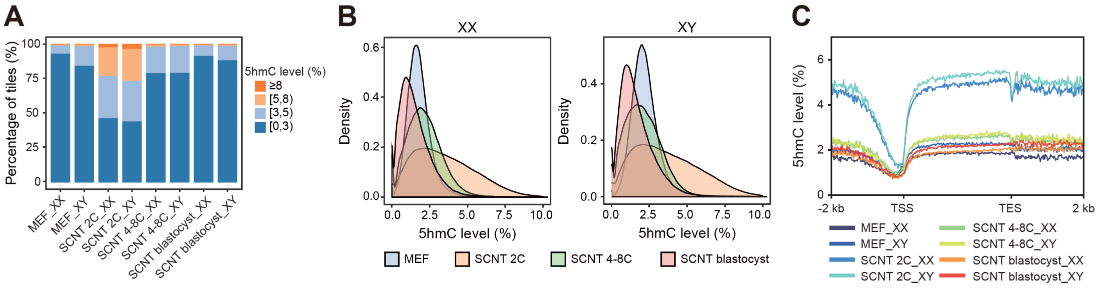

Figure S2. Intensity of 5hmC level across the genome during SCNT embryonic development.

A, Proportion of 10-kb tiles with 5hmC levels ≤3%, 3–5%, 5–8%, and ≥8% across the genomes of MEF and SCNT embryos of various developmental stages.

B, Density plots showing distribution of 5hmC levels in MEF and SCNT embryos (left, female embryos; right, male embryos).

C, Average 5hmCpG levels across gene bodies and flanking regions (±2 kb) in MEF and SCNT embryos. TSS, transcription start site; TES, transcription end site.

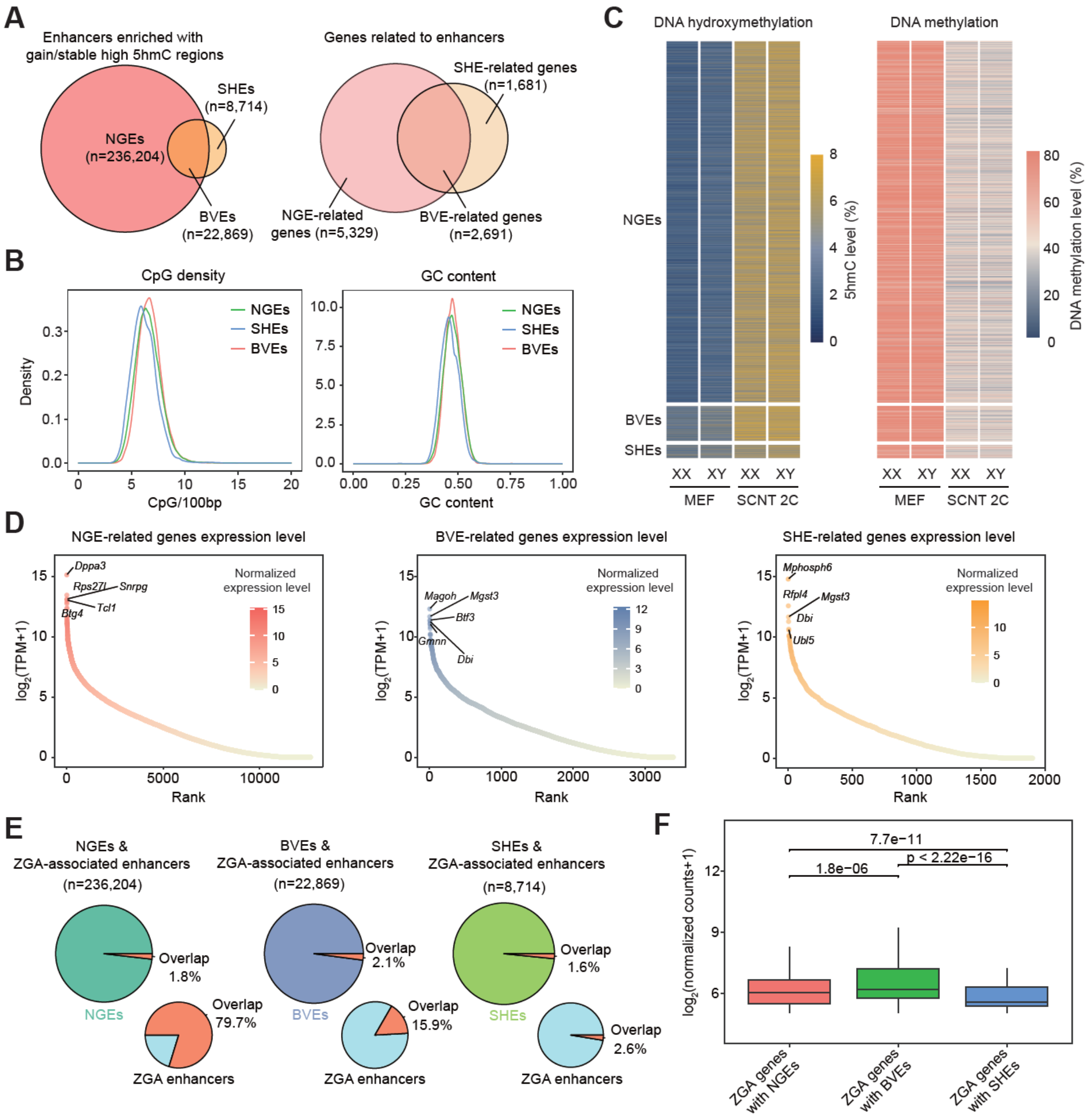

Figure S3. Comparative analysis of enhancers enriched with newly generated or stably high 5hmCpGs.

A, Venn plot showing bivalent enhancers enriched with both newly generated and stable high 5hmC regions (left) and the related genes (right).

B, Density plot analyzing the sequence properties (CpG density, left; GC content, right) of 3 types of enhancers.

C, Heatmaps displaying the 5hmC (left) and DNAme level alteration of NGEs, BVEs and SHEs during SZT.

D, Expression level of NGE, BVE and SHE-related genes at 2-cell stage. Genes were sorted by expression level rankings from high to low.

E, Pie charts showing the overlap of NGEs, BVEs, and SHEs with ZGA enhancers. Large pies indicate the proportion of ZGA enhancers overlapping each category; small pies show the proportion of each enhancer category within known ZGA enhancers.

F, Boxplot illustrating the expression level of ZGA genes related to the three types of enhancers. Significance was examined by Student's t-test. (ZGA genes with NGEs, n = 5329; with BVEs, n = 2691; with SHEs, n = 1681)

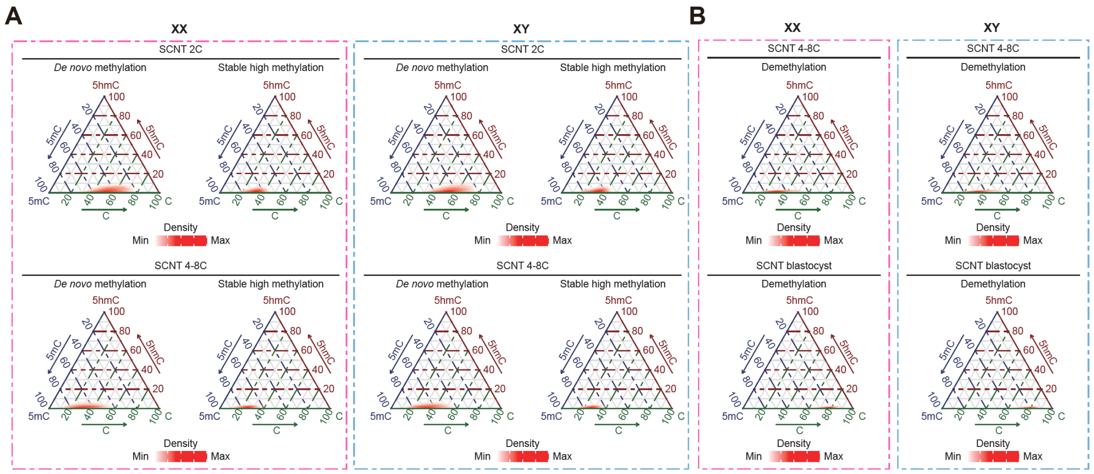

Figure S4. Correlation between 5hmC and 5mC during SCNT 2C to blastocyst.

A, Ternary plots showing the levels of cytosine, 5mC, and 5hmC in *de novo* methylation DMRs and maintenance regions during the 2-cell to 4/8-cell transition in SCNT embryos (left: female embryos; right: male embryos).

B, Ternary plots showing the levels of cytosine, 5mC, and 5hmC in maintenance regions during the 4–8-cell to blastocyst transition in SCNT embryos (left: female embryos; right: male embryos).

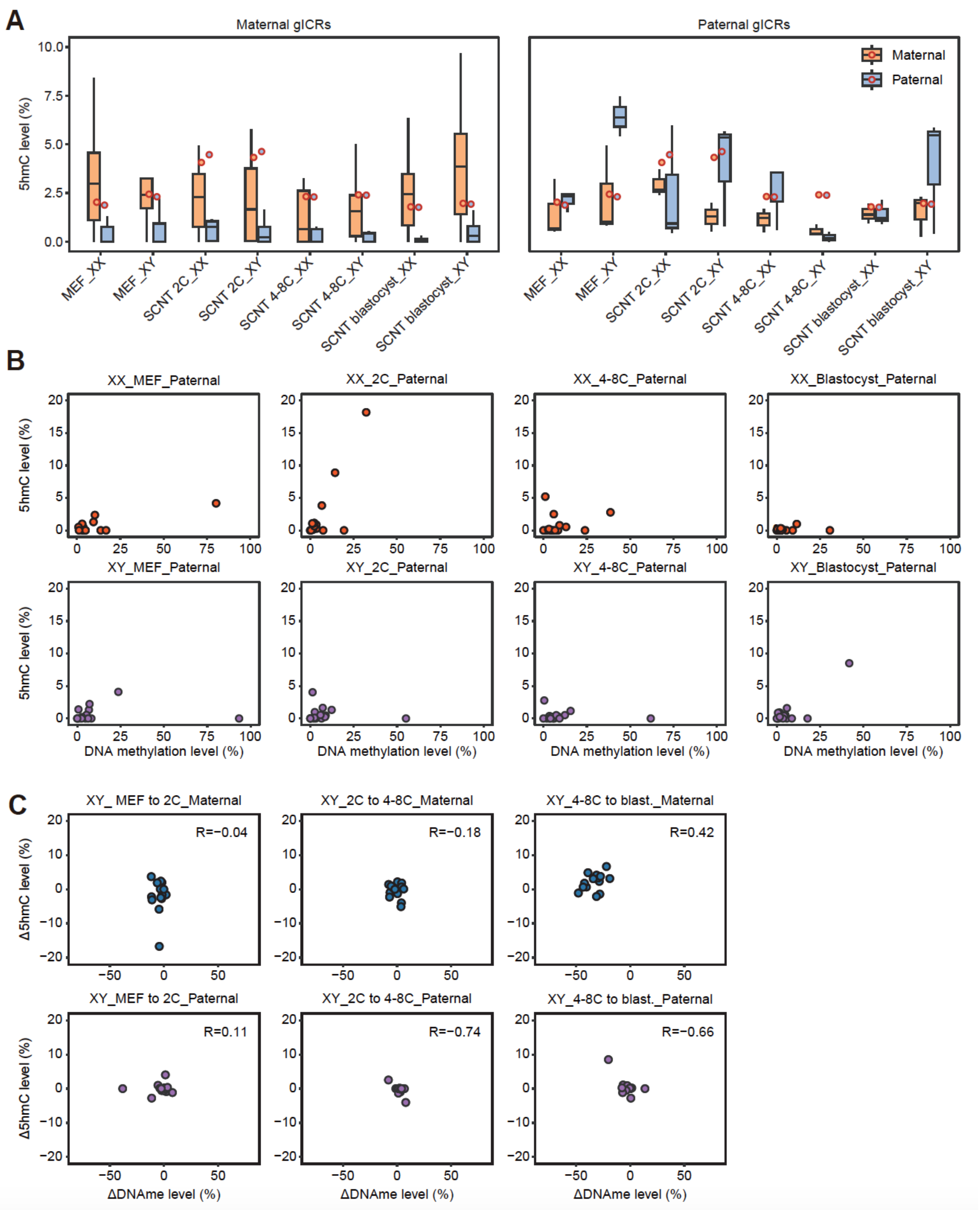

Figure S5. 5hmC dynamics in parental imprinted regions of SCNT embryos.

A, Box plots showing DNA methylation levels at maternal (left) and paternal (right) imprinting control regions (ICRs) in both parental genomes of SCNT embryos. Dots represent the global DNA methylation level of the same embryos (n = 2 biological replicates).

B, Dot plots showing DNA methylation and 5hmC levels at the same M_gICRs loci in the paternal genome. M_gICRs in female and male SCNT embryos are colored red and blue, respectively.

C, Dot plots showing DNA methylation and 5hmC level alterations between each 2 developmental stages at the same M_gICRs loci in the paternal genome. M_gICRs in female and male SCNT embryos are colored red and blue, respectively.

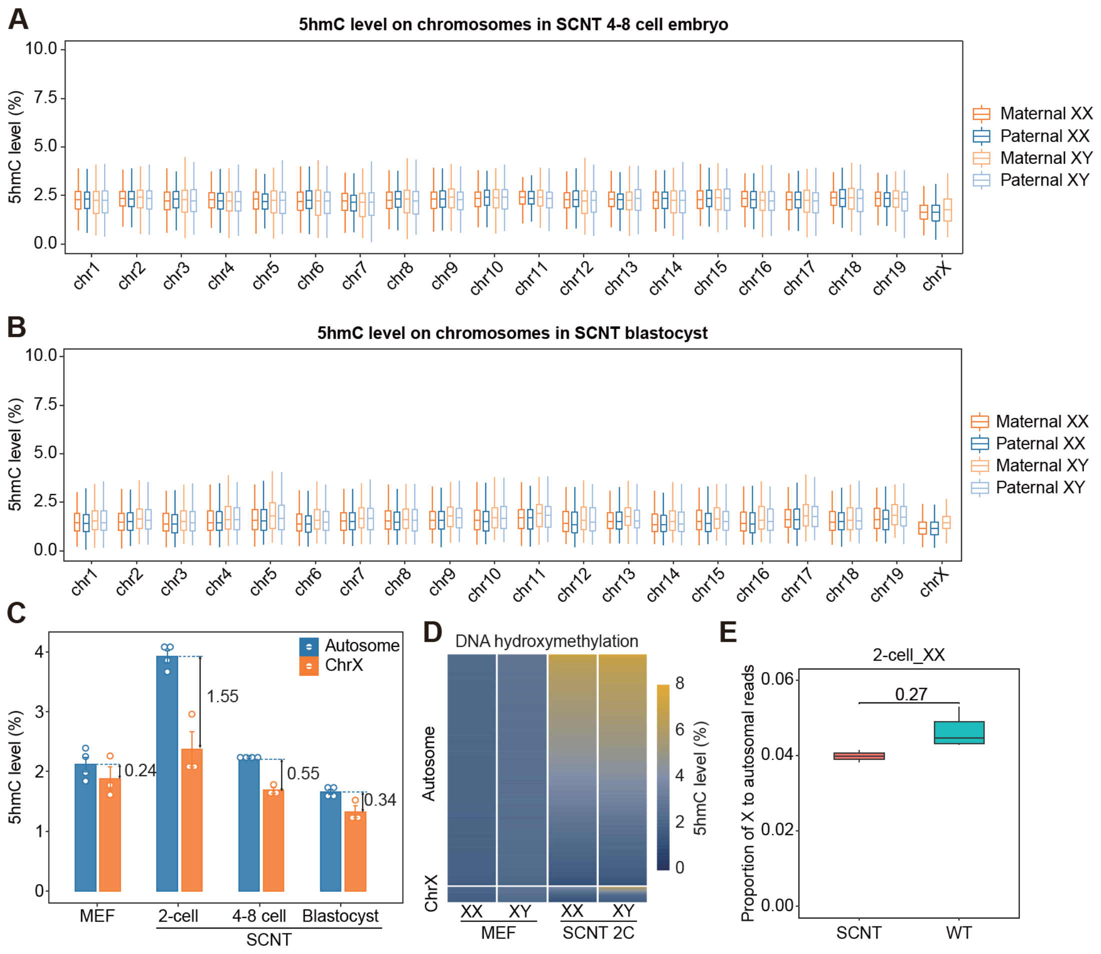

Figure S6. 5hmC level on autosomes and X chromosome during 4-8C to blastocyst.

A-B, Box plots showing 5hmC levels in 19 autosomes and X chromosome in maternal and paternal genome from SCNT 4-8 cell embryos (A) and blastocysts (B) of both genders (n = 2 biological replicates for each gender).

C, Bar plots quantitively display the 5hmC level difference between autosomes and X chromosome across developmental stages (n = 2 biological replicates for each gender at every stage).

D, Heatmap of DNA hydroxymethylation level changes in both autosomes and X chromosomes of both genders during SZT.

E, X/A ratio showing the relative transcriptive activity of X-linked genes in SCNT and WT 2-cell embryos (n = 2 biological replicates).

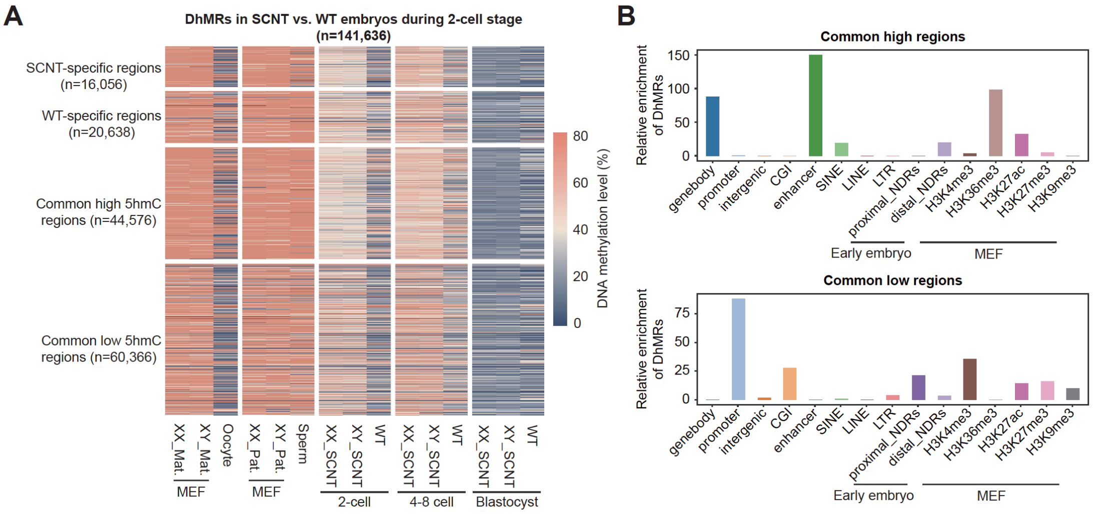

Figure S7. Methylation dynamics and commonly high/low 5hmC regions between WT and SCNT 2-cell embryos.

A, Heatmaps showing the DNA methylation dynamics of 4 categories of DhMRs/common regions between WT and SCNT embryos across developmental stages.

B, Enrichment analysis of common high (top) and common low (bottom) 5hmC regions across various genomic elements.

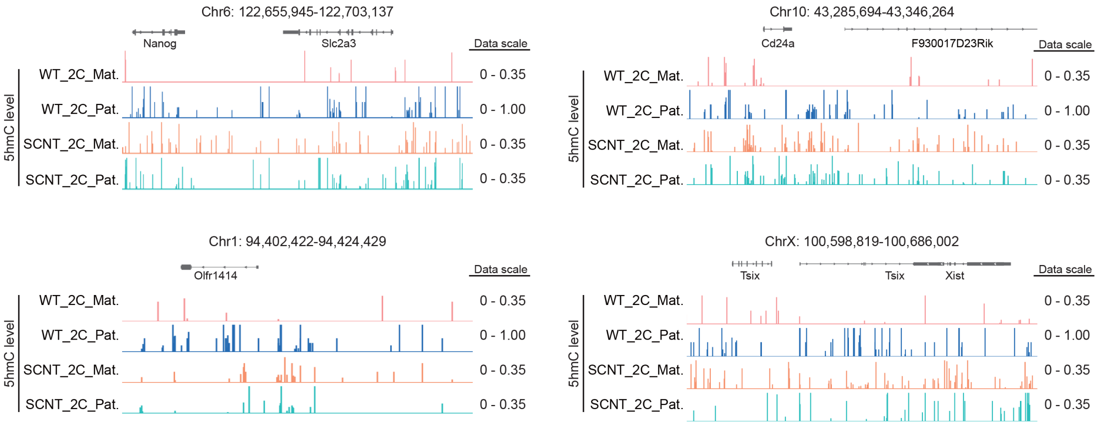

Figure S8. 5hmC distribution at representing sites of parental alleles in SCNT 2-cell embryos.

Track plot of representative 5hmC landscape on maternal and paternal genomes in WT and SCNT 2-cell embryos.

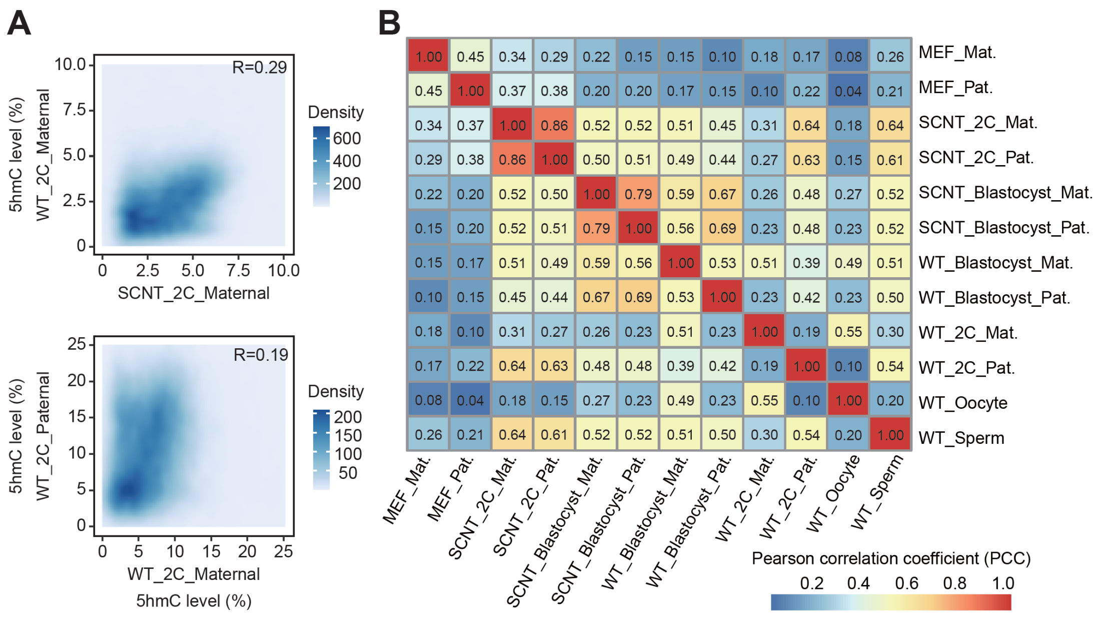

Figure S9. SCNT parental genome displays symmetrical 5hmC distribution at 2-cell stage.

A, Density plots comparing 5hmC levels in 100-kb genomic tiles between wild-type (WT) and SCNT 2-cell embryos.

B, Heatmaps showing correlation coefficients of 5hmC levels among maternal and paternal genomes of both WT and SCNT embryos.

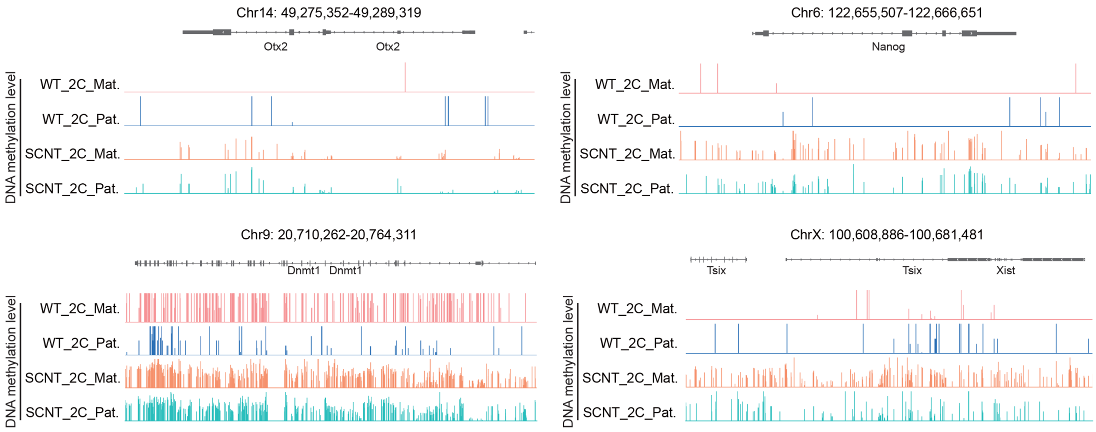

Figure S10. DNA methylation level at representing sites of parental alleles in SCNT 2-cell embryos.

Track plot of representative DNA methylation landscape on maternal and paternal genomes in WT and SCNT 2-cell embryos.

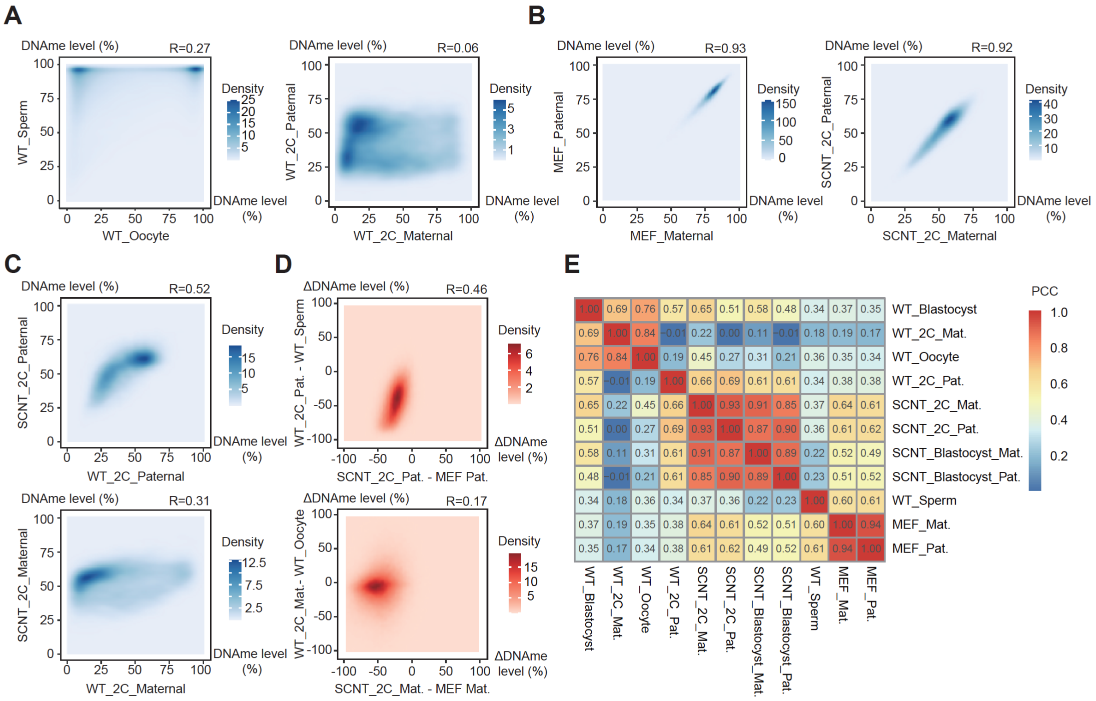

Figure S11. SCNT parental genome undergoes symmetrical DNA demethylation at 2-cell stage.

A, Density plots comparing DNA methylation levels in 100-kb genomic tiles between maternal and paternal genomes in donor cell (left) and SCNT 2-cell embryos (right).

B, Density plots comparing DNA methylation levels in 100-kb genomic tiles between germ cells (left) and between parental genomes of WT 2-cell embryos (right).

C, Density plots comparing DNA methylation levels in 100-kb genomic tiles of maternal (bottom) and paternal (top) genomes between WT and SCNT 2-cell embryos

D, Density plots comparing DNA methylation level changes during gametes/somatic cell-to-2-cell embryo transition in 100-kb genomic tiles of paternal (top) and maternal (bottom) genome between WT and SCNT embryos.

E, Heatmaps showing correlation coefficients of DNA methylation levels among maternal and paternal genomes in both WT and SCNT embryos.

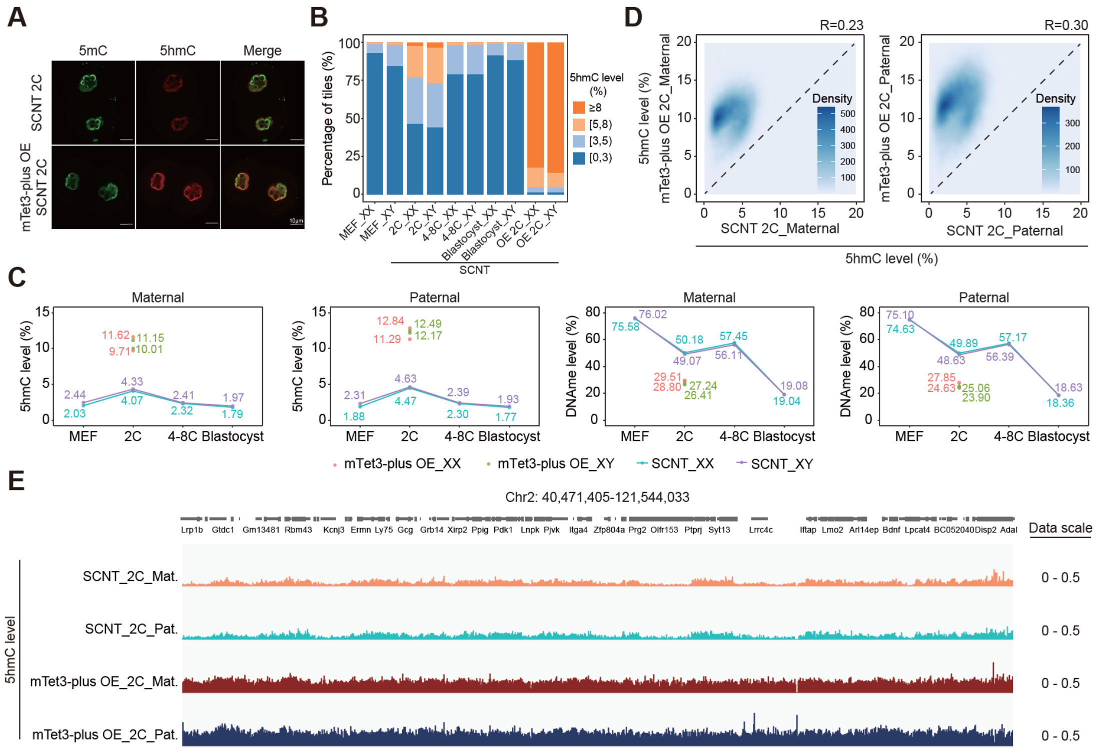

Figure S12. Excessive generation of 5hmC in mTet3-plus OE embryos.

A, Immunostaining of 5mC and 5hmC in SCNT 2-cell embryo injected with mTet3-plus mRNA compared to the control SCNT 2-cell embryo.

B, Proportion of 10-kb tiles with 5hmC levels ≤3%, 3–5%, 5–8%, and ≥8% across the genomes of SCNT 2-cell embryos with excessive 5hmC generation.

C, Dot plots showing 5hmCpG and DNA methylation levels in both parental genomes of Tet3-plus OE SCNT 2-cell embryos, compared with the dynamics in normal SCNT embryos (n = 2 biological replicates).

D, Density plots comparing 5hmC levels in 100-kb tiles of the maternal (left) and paternal (right) genomes between the Tet3OE group and the control group.

E, Track plot of representative 5hmC landscape on maternal and paternal genomes in mTet3-plus OE 2-cell embryos and the control SCNT embryos.

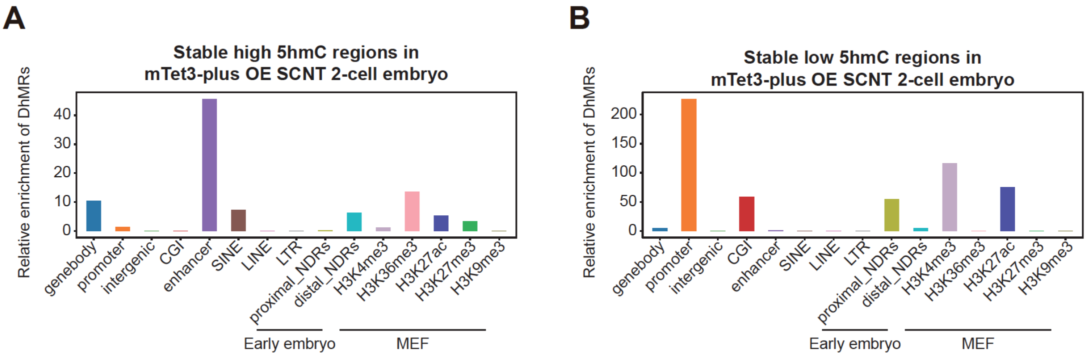

Figure S13. Enrichment of maintaining 5hmC regions in mTet3-plus OE 2cell embryos.

A, Bar plots showing enrichment scores of stable high 5hmC regions in mTet3-plus OE SCNT 2-cell embryos across different genomic elements.

B, Bar plots showing enrichment scores of stable low 5hmC regions in mTet3-plus OE SCNT 2-cell embryos across different genomic elements.

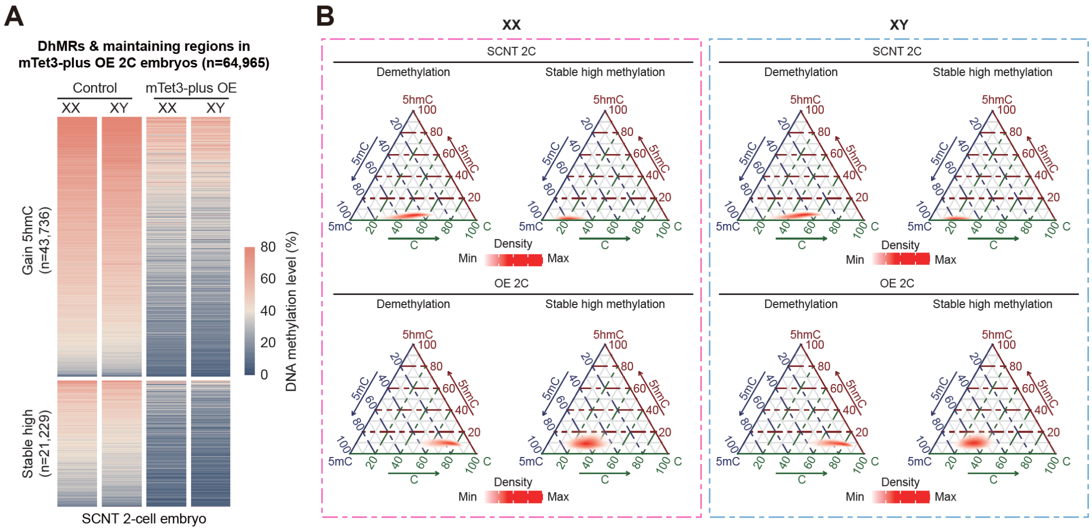

Figure S14. Correlations between excessive 5hmC and DNA hypo-methylation in SCNT embryos.

A, Heatmaps showing the 5mC levels in genomic regions identified in **Figure 7D** based on the 5hmC level change in mTet3-plus OE SCNT 2-cell embryos compared to SCNT 2-cell embryos.

B, Ternary plots showing the levels of cytosine, 5mC, and 5hmC in demethylation and maintenance regions of mTet3-plus OE SCNT 2-cell embryos compared to SCNT 2-cell embryos (left: XX embryos; right: XY embryos).

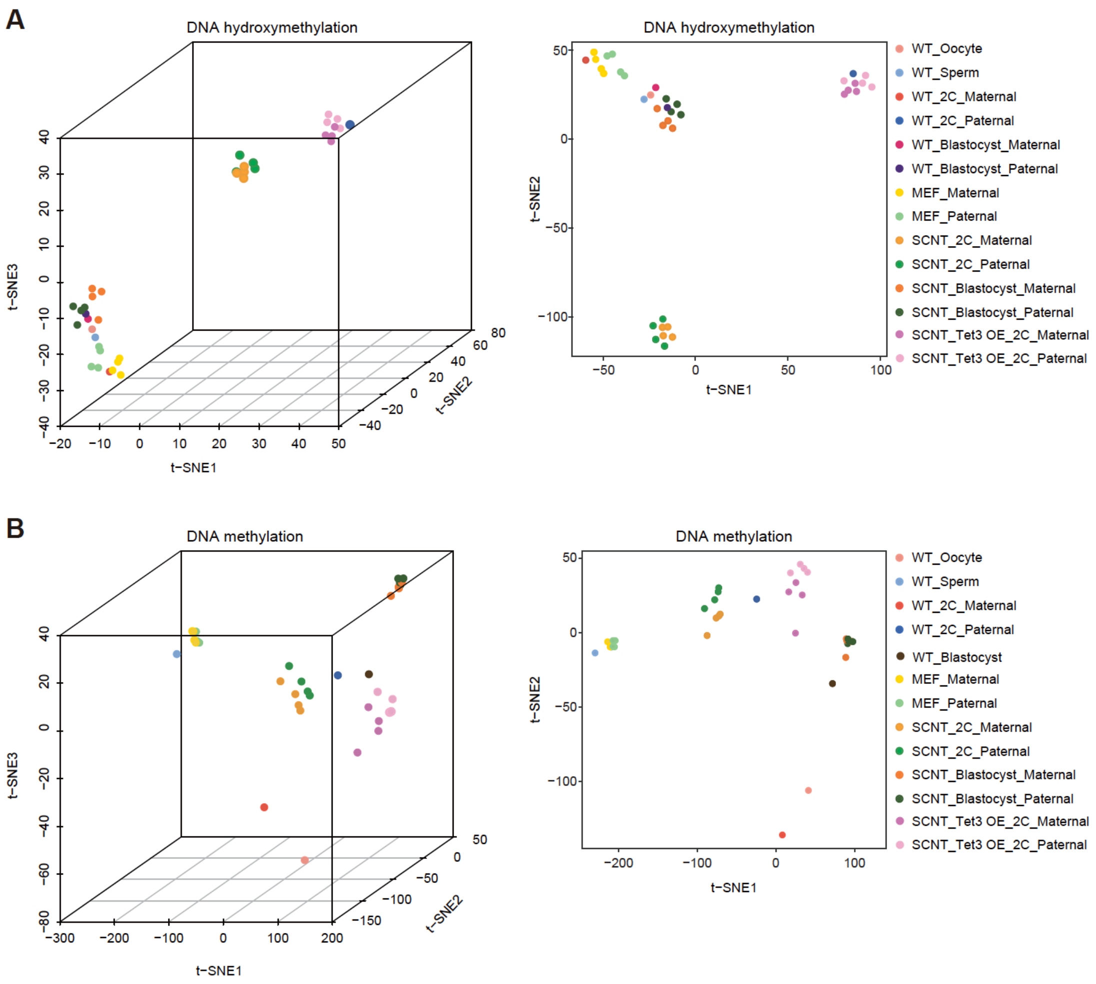

Figure S15. 5hmC & DNAme pattern of parental genome in SCNT embryos with elevated 5hmC.

A-B, Three-dimensional (left) and two-dimensional (right) t-SNE visualization of DNA hydroxymethylation (A) and DNA methylation (B) profiles in 100-kb tiles of the parental genomes in WT and SCNT embryos.

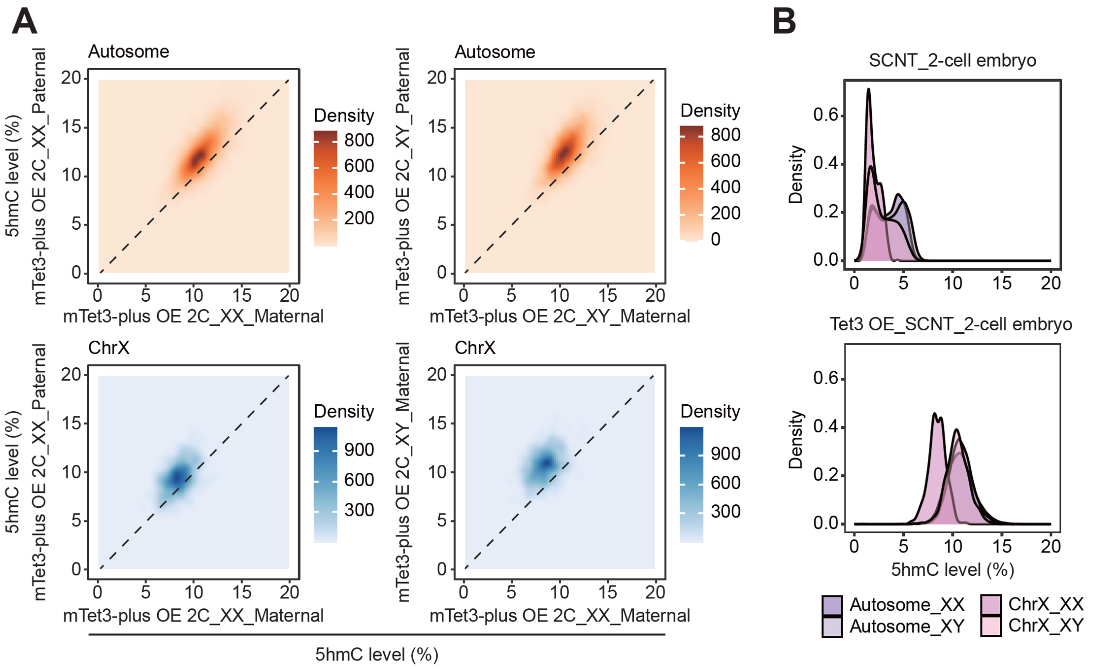

Figure S16. 5hmC level on X chromosome in mTet3-plus OE mouse embryos of both genders.

A, Density plots showing 5hmC levels in the maternal and paternal genomes on autosomes (top) and the X chromosome (bottom) in mTet3-plus OE SCNT 2-cell embryos.

B, Density plots showing the distribution of 5hmC levels on autosomes and the X chromosome in mTet3-plus OE SCNT 2-cell embryos and control SCNT 2-cell embryos.

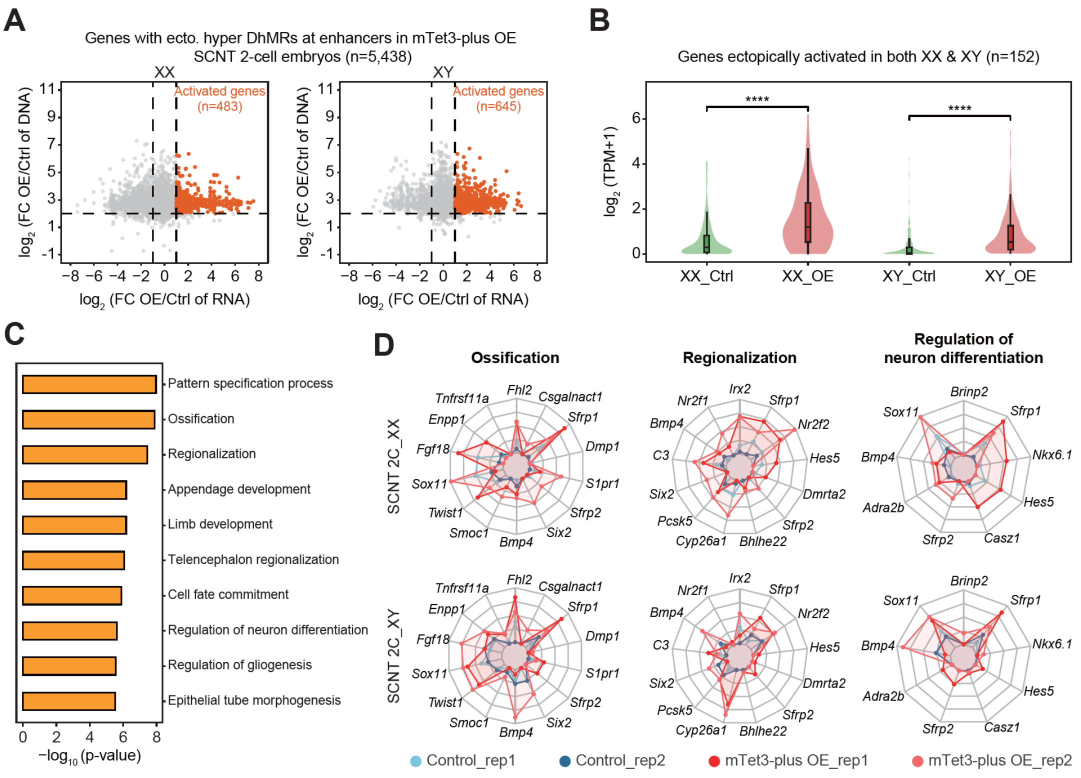

Figure S17. Function enrichment of genes with ectopical 5hmC generation at enhancers.

A, Scatter plots comparing mRNA expression changes (x-axis) and 5hmC/5mC level changes (y-axis) between mTet3-plus OE and control SCNT 2-cell embryos (left: XX embryos, right: XY embryos).

B, Violin plots showing the expression levels of ectopically activated genes in both XX and XY mTet3-plus OE SCNT 2-cell embryos.

C, Bar plots showing Gene Ontology (GO) enrichment of genes activated in mTet3-plus OE SCNT 2-cell embryos in Figure S15A.

D, Radar charts demonstrating the expression levels of genes associated with selected GO terms in Figure 8E and Figure S17C.

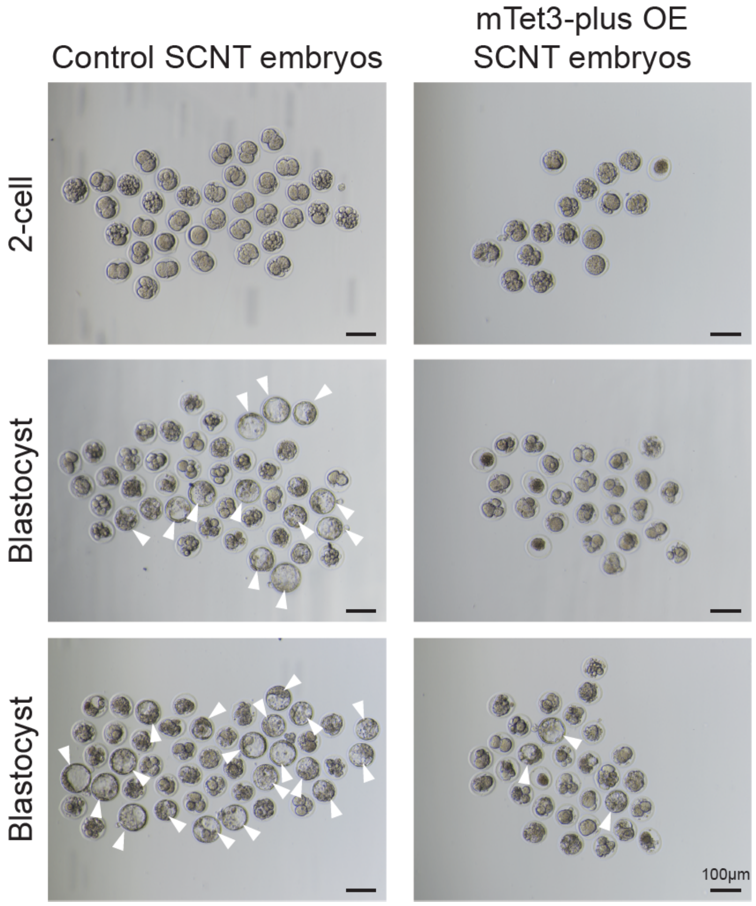

Figure S18. Images of embryonic development with excessive 5hmC generation. Representative images displaying the morphology of SCNT mouse embryos overexpressing mTet3-plus compared to the control SCNT embryos.
